# Mesenteric Adipose Tissue‐Derived *Klebsiella variicola* Disrupts Intestinal Barrier and Promotes Colitis by Type VI Secretion System

**DOI:** 10.1002/advs.202205272

**Published:** 2023-02-19

**Authors:** Junli Gong, Jing Yu, Shengmei Yin, Jia Ke, Jinjie Wu, Chen Liu, Zhanhao Luo, Wai Ming Cheng, Yaozu Xie, Yuan Chen, Zhen He, Ping Lan

**Affiliations:** ^1^ Department of Colorectal Surgery The Sixth Affiliated Hospital Sun Yat‐sen University Guangzhou Guangdong 510655 P. R. China; ^2^ Guangdong Provincial Key Laboratory of Colorectal and Pelvic Floor Diseases The Sixth Affiliated Hospital Sun Yat‐sen University Guangzhou Guangdong 510655 P. R. China; ^3^ Guangdong Institute of Gastroenterology Guangzhou Guangdong 510655 P. R. China; ^4^ School of Medicine Sun Yat‐sen University Guangzhou Guangdong 510275 P. R. China

**Keywords:** colitis, Crohn's disease, *Klebsiella variicola*, mesenteric adipose tissue, type VI secretion system

## Abstract

Mesenteric adipose tissue (MAT) in Crohn's disease (CD) is associated with transmural inflammation. Extended mesenteric excision can reduce surgical recurrence and improve long‐term outcomes, indicating that MAT plays an important role in the pathogenesis of CD. Bacterial translocation has been reported to occur in the MAT of patients with CD (CD‐MAT), but the mechanisms by which translocated bacteria lead to intestinal colitis remain unclear. Here it is shown that members of Enterobacteriaceae are highly enriched in CD‐MAT compared with non‐CD controls. Viable *Klebsiella variicola* in Enterobacteriaceae is isolated exclusively in CD‐MAT and can induce a pro‐inflammatory response in vitro and exacerbates colitis both in dextran sulfate sodium (DSS)‐induced colitis mice model and *IL‐10* ^−/−^ spontaneous colitis mice model. Mechanistically, active type VI secretion system (T6SS) is identified in the genome of *K. variicola*, which can impair the intestinal barrier by inhibiting the zonula occludens (ZO‐1) expression. Dysfunction of T6SS by CRISPR interference system alleviates the inhibitory effect of *K. variicola* on ZO‐1 expression and attenuated colitis in mice. Overall, these findings demonstrate that a novel colitis‐promoting bacteria exist in the mesenteric adipose tissue of CD, opening a new therapeutic avenue for colitis management.

## Graphical abstract

Enterobacteriaceae is highly enriched in the mesenteric adipose tissue from patients with Crohn's disease (CD‐MAT). *Klebsiella variicola* isolated from CD‐MAT promotes host intestinal inflammation through type VI secretion system (T6SS), which impairs intestinal barrier by inhibiting the expression of zonula occludens‐1 (ZO‐1). Dysfunction of T6SS alleviates the inhibitory effect of *K. variicola* on ZO‐1 expression and attenuates colitis in mice.

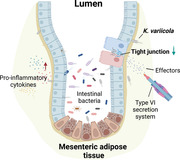

## 1 Introduction

Mesenteric adipose tissue (MAT) around the involved intestine in Crohn's disease (CD) form a special extra‐intestinal manifestation called “creeping fat.”^[^ ^1^ ^]^ It is topographically associated with the occurrence of transmural inflammation, fibrosis, and stricture and is independently associated with disabling course, bowel damage, and abdominal surgery.^[^ ^2^ ^]^ Therefore, MAT of patients with CD (CD‐MAT) has been regarded as a pathological feature of CD.^[^ ^3^ ^]^ Due to that extended mesenteric excision has been proven to reduce surgical recurrence and improve long‐term outcomes,^[^ ^4^, ^5^ ^]^ CD‐MAT has recently emerged as an independent risk factor for the pathogenesis of CD. However, how does CD‐MAT influence the pathogenesis of CD and the underlying mechanisms remain to be demonstrated.

An “outside‐in effect” has been proposed to be involved in MAT‐induced intestinal inflammation.^[^ ^6^ ^]^ Pro‐inflammatory cytokines, such as C‐reactive protein, resistin, leptin, and adiponectin, produced locally in MAT have been suggested to induce a systemic inflammatory response and intestinal colitis.^[^ ^6^, ^7^ ^]^ Considering the initial of inflammatory response in CD‐MAT, bacteria existed in MAT are of great concern. Currently, there is growing evidence that bacteria translocated in MAT occurs in host intestinal inflammation.^[^ ^6^, ^8^, ^9^, ^10^ ^]^ Ha et al. (2020) reported that specific immune responses elicited by the translocated bacteria (*Clostridium innocuum*) could induce the formation of “creeping fat.”^[^ ^9^ ^]^ Additionally, using multidimensional data, we previously reported that the existence of the MAT microbiome contributed to alterations in the metabolic and transcriptional characteristics of CD‐MAT, and certain isolated pathogenic bacteria in MAT could exacerbate colitis in mice.^[^ ^10^ ^]^ However, the mechanism by which the translocated bacteria promote intestinal colitis remain unclear.

Transmural inflammation commonly occurs during CD development.^[^ ^11^ ^]^ Expansion of Enterobacteriaceae has been regarded as a dominant dysbiosis characteristic in inflammatory bowel disease (IBD) patients.^[^ ^12^ ^]^ Studies have revealed the existence of elevated numbers of mucosa‐associated *Escherichia coli* (*E. coli*) in IBD patients,^[^ ^13^ ^]^ and an enrichment of adherent invasive *E. coli* (AIEC) strains in the ileac mucosa of patients with CD.^[^ ^14^ ^]^ Bacteria in MAT has been proposed to be originally translocated from the intestinal lumen.^[^ ^9^ ^]^ Hence, we proposed that MAT may act as another hidden place for CD‐related pathobionts. The epithelial barrier, maintained by the regulation of tight junctions (TJs) and adherens junctions (AJs), plays a critical role in protecting the body from exogenous elements including pathogenic bacteria,^[^ ^15^, ^16^ ^]^ and breakdown of this barrier is a hallmark of IBD.^[^ ^17^, ^18^ ^]^ Intestinal pathobionts (e.g., *Campylobacter jejuni* and *Clostridium difficile)* have been shown to destabilize host intestinal mucosal barrier by secreting enzymes or toxins that cleave the mucus or directly disrupt tight junctions in the underlying epithelial cells.^[^ ^19^, ^20^ ^]^ Secretion system is widespread in intestinal associated pathobionts, which dedicated to delivery toxin into prokaryotic and eukaryotic cells.^[^ ^21^ ^]^ Recent studies have shown that the type VI secretion system (T6SS) is involved in suppressing host immune response and reorganizing the actin cytoskeleton within host cells.^[^ ^22^, ^23^ ^]^ Actin cytoskeleton regulates and disrupts TJs and AJs that maintain epithelial barrier.^[^ ^24^ ^]^ However, it is unknown whether T6SS is involved in the pathogenesis of CD.

Here, we analyzed the bacterial community structure in MAT and identified the CD‐MAT specific bacteria by 16S rRNA gene sequencing and culture‐omics. The role of our identified bacteria in inducing intestinal barrier dysfunction and promoting inflammation was examined in vitro and in vivo. We reported that the pro‐inflammatory and barrier disruption roles of our candidate bacteria were mediated by T6SS. Additionally, we also demonstrated that T6SS was highly expressed in the fecal microbiome of patients with CD.

## 2 Results

### 2.1 Members of Enterobacteriaceae Are Enriched in the Mesenteric Adipocytes Tissue (MAT) of CD Patients Compared to the Non‐CD Controls

**Figure 1:**
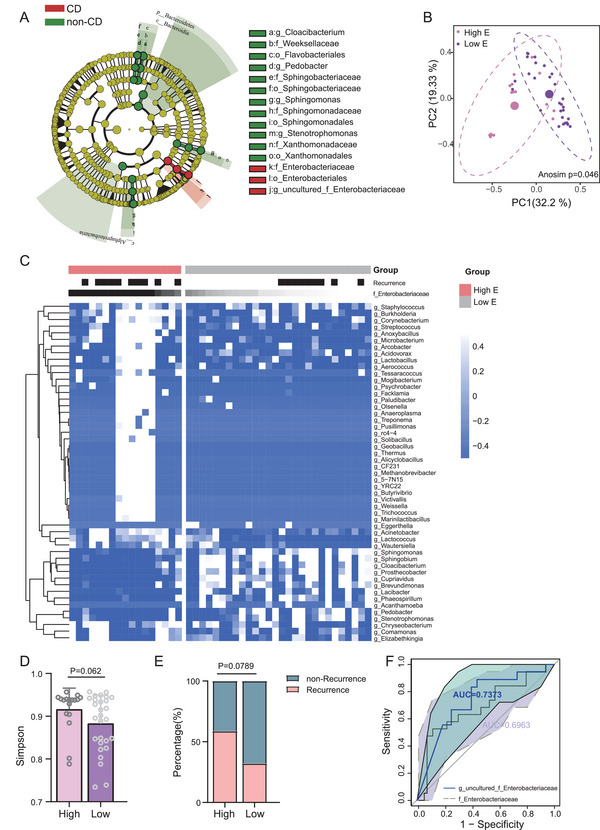
Enterobacteriaceae are enriched in the mesenteric adipocytes tissue (MAT) of CD patients compared to the non‐CD controls. A) Differentially distributed taxa in MAT of CD patients (red) and the non‐CD controls (green) by linear discriminant analysis effect size (LEfSe) analysis (LDA ≥ 4). B) PCoA based on weighted UniFrac analysis of bacterial communities in MAT of CD patients with high E and low E. Analysis of similarities (Anosim) was employed to assess statistical significance between high E and low E. C) Heat map analysis revealed the differential distributed genera in MAT between CD patients with High E and Low E. D) Simpson index showed the alpha diversity between groups high E and low E. E) The distribution of patients with recurrence and non‐recurrence in groups High E and Low E. F) Receiver operating characteristic (ROC) analysis using f_Enterobacteriaceae and uncultured_f_ Enterobacteriaceae to classify patients with CD and the non‐CD controls. High E: high abundance of Enterobacteriaceae; Low E: low abundance of Enterobacteriaceae. Error bars ± SEM. *p < 0.05; **p < 0.01; ***p < 0.001; Mann–Whitney U‐test.

We performed linear discriminant analysis effect size (LEfSe) analysis (LDA ≥ 4) to identify the differentially abundant taxa in the MAT of CD at multiple levels. It was observed that Enterobacteriales, Enterobacteriaceae (a family in Enterobacteriales), and uncultured_f_Enterobacteriaceae were consistently significantly enriched in the mesenteric tissue of patients with CD (**Figure** **1**A), indicating that they may be closely associated with CD pathogenesis. To evaluate the important role of Enterobacteriaceae in the MATs of CD, we classified our recruited patients with CD into high Enterobacteriaceae (high‐E) and low Enterobacteriaceae (low‐E) groups according to the median value of the abundance of Enterobacteriaceae in MAT. We observed that the bacterial community in MATs with a high E exhibited distinct microbiotic characteristics compared to those with a lower abundance of Enterobacteriaceae (Figure 1B). Furthermore, the differentially distributed microbial community was characterized between patients with high and low E, which showed that an increase in *Streptococcus*, *Burkholderia*, *Pseudomonas*, *Acinetobacter*, *Geobaccillus*, and *Thermus* was associated with Enterobacteriaceae accumulation in CD‐MAT (Figure 1C; Figure S1, Supporting Information). Additionally, we found that patients with a higher Enterobacteriaceae abundance had a lower bacterial diversity (Figure 1D), which might be resulted from increased distribution of a dominant group of bacteria. Moreover, an increased proportion of CD patients who experienced recurrence was observed in the high‐E group (Figure 1E). In addition, to explore Enterobacteriaceae's ability to distinguish CD from non‐CD, receiver operator characteristic (ROC) analysis was used to calculate the area under curve (AUC). The results showed an AUC value of 0.6963 for Enterobacteriaceae, while the uncultured_f_Enterobacteriaceae showed an AUC value of 0.7373, indicating that specific members of the family Enterobacteriaceae contributed to the bacterial configuration in CD‐MAT (Figure 1F). Overall, these data demonstrated that mesenteric tissues of patients with CD harbored a significantly differentially distributed microbiota, and enrichment of specific members of Enterobacteriaceae was a determinant characteristic in CD‐MAT.

### 2.2 Viable *Klebsiella variicola* in Enterobacteriaceae Is Exclusively Isolated from the Mesenteric Tissue of CD Patients

**Figure 2:**
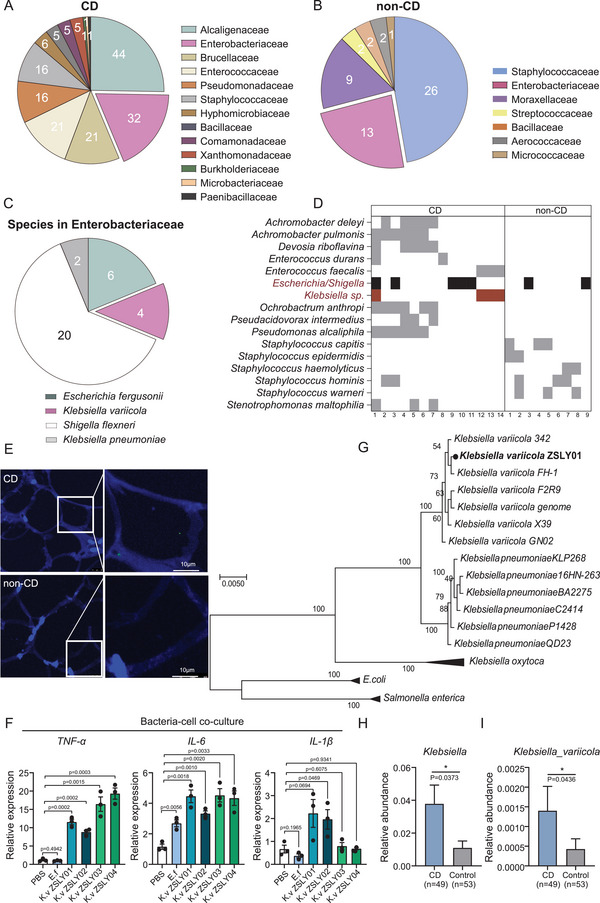
*Klebsiella variicola* in family Enterobacteriaceae is exclusively isolated from the MAT of CD patients. A,B) A pie chart illustrating the number of isolates belonging to each bacterial family in MAT of CD patients and the non‐CD control. C) The specific species in the family Enterobacteriaceae. D) Cultivable bacteria recovered from MAT of CD and non‐CD (at the genus level). In each group, bacteria isolated from more than one specimen are shown. Each column represents the cultivable bacteria community in an individual patient. Members belonging to the family Enterobacteriaceae are shaded black, while members exclusively isolated from CD MAT are shaded red. E) Detection of *Klebsiella* spp. in MAT from CD patients and the non‐CD controls by fluorescence in situ hybridization (FISH). Sections of MAT were stained with DAPI (blue), *Klebsiella* spp. (green), (scale bar = 10 µm). F) Four strains of *K. variicola* (*K. variicola* ZSLY01, *K. variicola* ZSLY02, *K. variicola* ZSLY03, *K. variicola* ZSLY04) elicit inflammatory responses toward IEC‐6 (rat intestinal epithelial cells) (Unpaired Student's *t*‐test). G) Phylogenetic analysis by maximum likelihood for identification of the isolated *Klebsiella* spp., based on conserved functional genes derived from whole‐genome sequencing. (H&I) The distribution of *Klebsiella* spp. and *K. variicola* in the gut microbiome of patients with CD using published data (publicly available database (PRJEB15371) (Mann–Whitney U‐test). *p < 0.05; **p < 0.01; ***p < 0.001; ****p < 0.0001.

To investigate the viable translocated members of Enterobacteriaceae in the mesenteric tissues, we cultured 23 specimens (14 CD and 9 non‐CD) under anaerobic and aerobic conditions and picked 229 colonies (174 from CD and 55 from control) with different colony appearances on different culture plates. 16S rRNA gene sequencing revealed that these colonies were classified into 17 families (**Figure** **2**A), with 13 families identified in the mesenteric tissue of CD and seven in the non‐CD control (Figure 2B). Totally, 32 colonies belonging to Enterobacteriaceae were identified from the MAT of patients with CD, which were categorized into four taxa groups (*Escherichia fergusonii, Klebsiella variicola, Shigella flexneri*, and *Klebsiella pneumoniae*) (Figure 2C). As shown in Figure 2D, *Escherichia/Shigella* were repeatedly isolated from the mesenteric tissue of CD and the control group, whereas *Klebsiella* spp. were exclusively isolated from the mesenteric tissue of CD (Figure 2D). Observation of *Klebsiella* spp. in CD‐MAT was verified by fluorescence in situ hybridization (FISH) using *Klebsiella*‐specific probes under sterile conditions (Figure 2E).

The colitogenic roles of *Klebsiella pneumoniae* and *Klebsiella oxytoca* in the initiation and perpetuation of colitis have been revealed,^[^ ^25^, ^26^, ^27^ ^]^ however, the pro‐inflammatory role of *K. variicola* was rarely mentioned.^[^ ^28^ ^]^ To determine the pro‐inflammatory effect of our 4 isolated strains of *K. variicola* (named as *K. variicola* ZSLY01, *K. variicola* ZSLY02, *K. variicola* ZSLY03, and *K. variicola* ZSLY04), a bacteria‐cell (intestinal cell line IEC6) co‐culture experiment was carried out. The result showed that, despite the extent of their pro‐inflammatory ability differed with each other, 4 strains of *K. variicola* could respectively evoke a strong pro‐inflammatory response toward IEC‐6 cell, in comparison to a commensal bacterial strain of *E. fergusonii* (Figure 2F).^[^ ^10^ ^]^ Of them, *K. variicola* ZSLY01 was chosen as a representative strain for our subsequent in vitro and in vivo study (all *K. variicola* mentioned in the following context refers to *K. variicola* ZSLY01 unless otherwise specified). Whole‐genome sequencing of *K. variicola* was performed, phylogenetic identity of *K. variicol*a was confirmed again based on a set of conserved functional genes (Figure 2G). Functional analysis of the genomic genes in *K. variicola* revealed 58 genes for cell motility and 135 genes for lipid transport and metabolism (Figure S2A, Supporting Information). Additionally, genes involved in protecting against oxidative damage (superoxide dismutase, nitrate reductase, thioredoxin reductase, and peroxiredoxin) and promoting bacterial survival and proliferation in the host tissue (arginase) were also observed in *K. variicola's* genome (Figure S2B, Supporting Information), suggesting that *K. variicola* could be available for survival in MAT.^[^ ^29^ ^]^ To show *K. variicola*’s ability to invade adipose tissue, we carried out a bacteria‐cell co‐culture assay in vitro. As expected, *K. variicola* could successfully invade into 3T3L1 cell (mice preadipocytes cell line) (Figure S2C, Supporting Information). The pro‐inflammatory role of *K. variicola* toward preadipocytes cells (3T3L1) was also confirmed (Figure S2D, Supporting Information). The distribution of *K. variicola* in MAT of our recruited cohort was determined by absolute (or standard curve) quantitative PCR. *K. variicola* was positively detected in 16 out of 47 (34.04%) CD‐MAT samples, in comparison to 4 out of 18 (22.22%) in the non‐CD‐MAT. The distribution of *K. variicola* in CD‐MAT was in the range of 14–66 537 CFU mg^−1^, with a median abundance being 65.5 CFU mg^−1^ (Table S4, Supporting Information). In addition, we collected 16 CD and 8 non‐CD terminal ileum mucosa to detect the distribution of *K. variicola*. The results showed that *K. variicola* was positively detected in 7 out of 16 (43.75%) CD‐intestinal tissues, in comparison to 1 out of 8 (12.5%) in the non‐CD‐intestinal tissues. The distribution of *K. variicola* in terminal ileum mucosa from CD patients was in the range of 9–13 718 CFU mg^−1^, with a median abundance being 112 CFU mg^−1^ (Table S5, Supporting Information). Besides, we also confirmed that a significant enrichment of *Klebsiella* and especially *K. variicola* existed in the fecal samples of patients with CD (Figure 2H,I), which was re‐analyzed from a fecal metagenomic sequencing data in a publicly available database (PRJEB15371).^[^ ^30^ ^]^ Taken together, these results demonstrated the existence of a specific viable translocated Enterobacteriaceae member—*K. variicola*, in the mesenteric tissue, and that colonization of *K. variicola* in CD‐MAT might be associated with the bacterial translocation from gut lumen. Notably, *K. variicola* can invade preadipocytes and act as an inflammation inducer in epithelial and preadipocyte cells.

### 2.3 *K. variicola* Exacerbates Colitis in Mice

**Figure 3:**
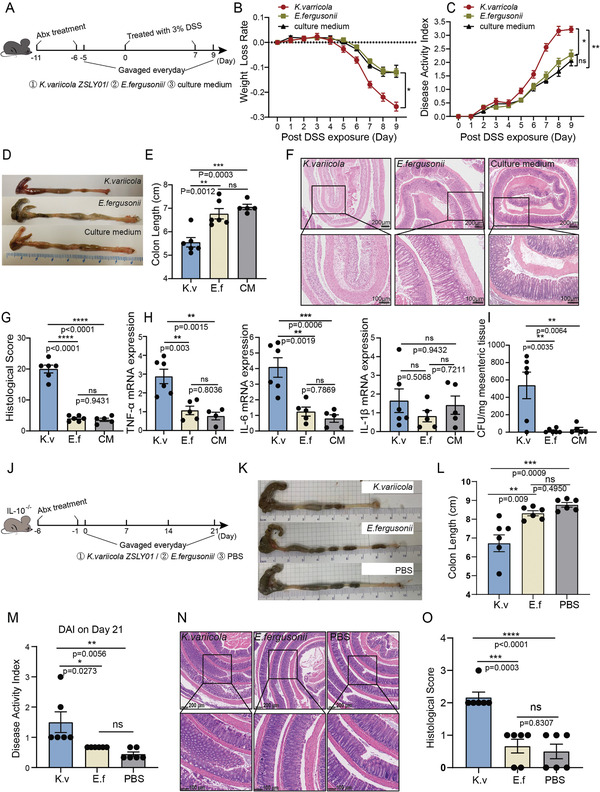
*K. variicola* exacerbates colitis in mice. A) mice experiment design: SPF C57BL/6 male mice (*n* = 5, 6) were treated with an antibiotic cocktail for 5 days. Daily gavage of bacteria (*K. variicola* ZSLY01 or *E. fergusonii*, 1×10^9^ CFU/mouse) or the culture medium for 14 days. To induce colitis, 3% dextran sodium sulfate (DSS) instead of drinking water was given to mice from day 0 to day 7. B) Weight loss rate and C) disease activity index (DAI) after 3% DSS treatment. D) Representative colons, E) colon length, F) representative colonic histological images (scale bar = 200 µm and 100 µm), and G) colonic histological score of mice treated by *K. variicola* ZSLY01, *E. fergusonii*, and the culture medium. H) Expression of pro‐inflammatory cytokines (*TNF‐a*, *IL‐6*, and *IL‐1β*) in the mice intestines. I) Detection of translocated *K. variicola* in mice MAT. J) Mice experiment design: SPF IL10^−/−^ mice (*n* = 6) were treated with an antibiotic cocktail for 5 days and daily bacteria (*K. variicola* ZSLY01 or *E. fergusonii*,1×10^9^CFU/mouse) or PBS gavage started on day 0 and continued until euthanized on day 21. K,L) Representative colons and colon length. M) Disease activity index (DAI) on day 21. N) Representative colonic histological images (scale bar = 200 µm and 100 µm) and colonic histological score from IL‐10^−/−^ mice treated by *K. variicola* ZSLY01, *E. fergusonii*, and PBS. O) Colonic histological score of IL‐10^−/−^ mice treated by *K. variicola* ZSLY01, *E. fergusonii*, and PBS. K.v, *K. variicola* ZSLY01; E.f, *E. fergusonii*. CM: culture medium. Error bars ± SEM. *p < 0.05; **p < 0.01; ***p < 0.001; ****p < 0.0001; one‐way ANOVA with Tukey's multiple comparison test. Each dot represents an individual mouse; two‐way ANOVA with Tukey's multiple comparison test (B, C).

To further evaluate the pathogenic ability of *K. variicola* in vivo (**Figure** **3**A), we colonized antibiotic‐treated specific pathogen‐free (SPF) mice with *K. variicola*, while *E. fergusonii* or the culture medium. Expectedly, *K. variicola*‐treated mice had exacerbated DSS‐induced colitis, as indicated by a sharp weight loss (Figure 3B), heightened disease activity index (DAI) (Figure 3C), and shortened colon (Figure 3D,E). Colonic inflammation was verified by histological assessment, as shown by increased mucosal erosion, crypt destruction, and inflammatory cell infiltration in *K. variicola*‐treated group in comparison with mice treated with *E. fergusonii* and the culture medium control (Figure 3F,G). In accordance with the histological alteration, we observed a significant increase in colonic mRNA expression of TNF‐*α* and IL‐6 in mice colonized with *K. variicola* (Figure 3H). Meanwhile, translocation of *K. variicola* in mouse mesenteric tissue was examined, and *K. variicola* was positively detected in 5/6 of the mice, with a range of 128–873 CFU mg^−1^ tissue (Figure 3I).

Unlike wild‐type C57BL/6 mice, the interleukin‐10 deficient (IL‐10^−/−^) mice spontaneously developed chronic enterocolitis similar with chronic IBD in humans, when exposed to normal commensal bacteria.^[^ ^31^ ^]^ Thus, to better understand the inflammation‐promoting role of *K. variicola*, an IL‐10‐deficient mouse model was used in this study (Figure 3J). Consistently, a significant colitis‐promoting role of *K. variicola* was identified, as evidenced by a significant shortening of colon length (Figure 3K,L) and a heightened DAI (Figure 3M). Meanwhile, histological features of inflammation characterized by mucosal thickening and aggravation of colonic inflammation were also observed in *K. variicola* treated mice, but not in those treated with commensal bacteria *E. fergusonii* or PBS (Figure 3N,O).

Additionally, to determine whether gut colonization by *K. variicola* is sufficient to exacerbate intestinal inflammation in the presence of gut microbiota, we colonized SPFwild‐type C57BL/6 mice with *K. variicola* in the DSS‐induced colitis model (Figure S3A, Supporting Information). The results showed that the gavage of *K. variicola* in SPF mice had no significant difference in weight loss rate compared to mice in PBS control group (Figure S3B, Supporting Information). However, an increased degree of hematochezia was obviously observed in *K. variicola*‐treated mice (data not shown), resulting in an increased DAI in *K. variicola*‐treated group (Figure S3C, Supporting Information). Additionally, the shortening of colon length (Figure S3D,E, Supporting Information) and increased *TNF‐α* expression indicated that *K. variicola* still had a significant pathogenic effect on SPF mice (Figure S3F, Supporting Information). Collectively, our results showed that mesenteric‐derived *K. variicola* exacerbated colitis in mice.

### 2.4 *K. variicola* Impairs the Intestinal Barrier Function

**Figure 4:**
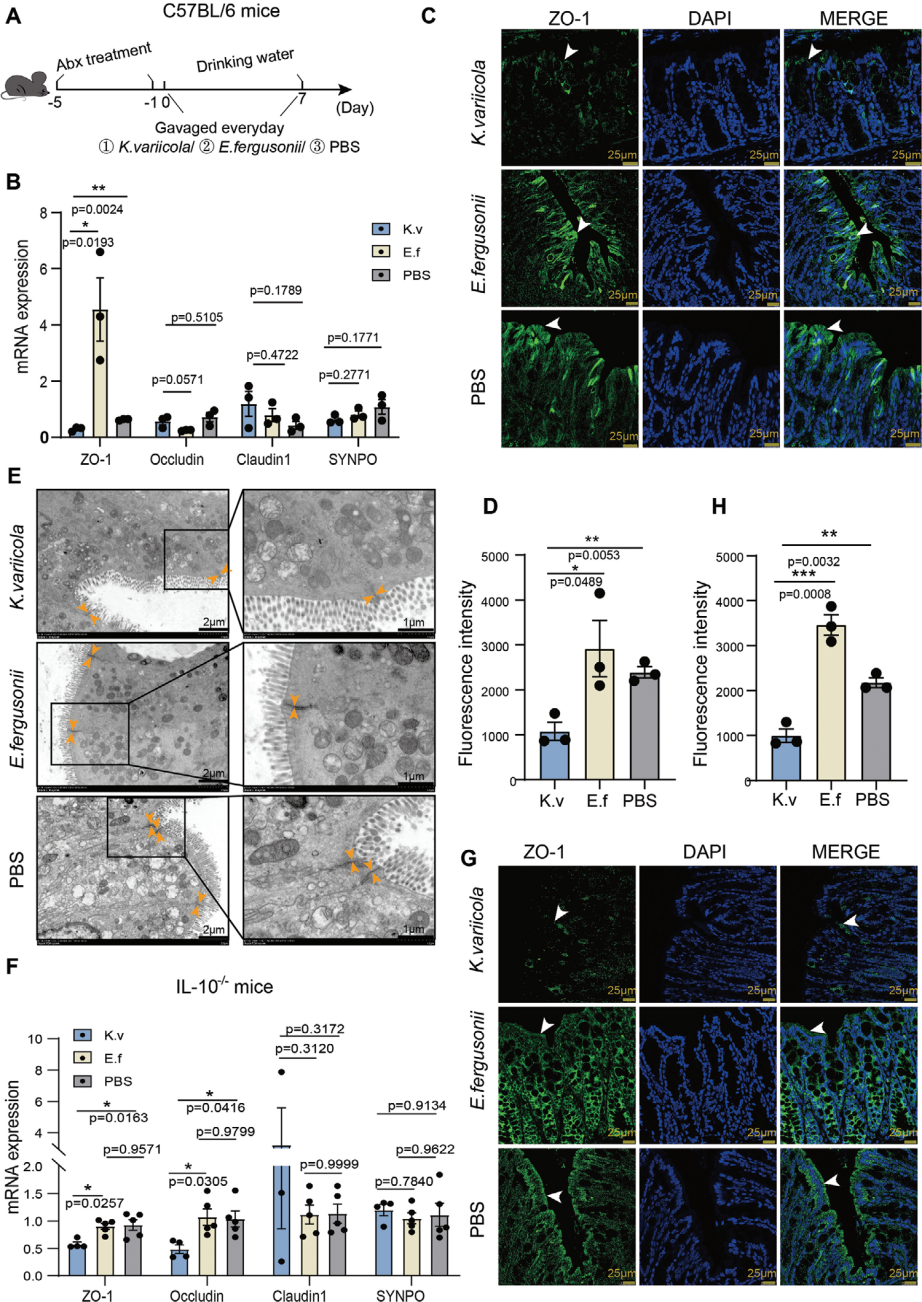
*K. variicola* impairs the intestinal barrier function. A) mice experiment design. SPF C57BL/6 male mice (*n* = 5) were treated with an antibiotic cocktail for 5 days. Daily gavage of bacteria (*K. variicola* ZSLY01 or *E. fergusonii*, 1×10^9^ CFU/mouse) or PBS started on day 0 and continued for 7 consecutive days. B) Expression of tight junctions in mice colons from *K. variicola* ZSLY01*, E. fergusonii*, or PBS treated mice. C,D) Immunofluorescent staining and quantification of the expression of ZO‐1 in *K. variicola* ZSLY01, *E. fergusonii*, or PBS treated mice colon tissues (scale bar = 25 µm). E) Evaluation of the expression of tight junctions by electron microscopy in *K. variicola* ZSLY01, *E. fergusonii*, or PBS treated mice colon tissues. F) Expression of tight junctions in *K. variicola* ZSLY01, *E. fergusonii*, or PBS treated IL10^−/−^ mice (*n* = 4–5) colon tissues. G,H) Immunofluorescent staining and quantification of the expression of ZO‐1 in *K. variicola* ZSLY01, *E. fergusonii*, or PBS treated IL10^−/−^ mice colon tissues (scale bar = 25 µm). K.v, *K. variicola* ZSLY01; E.f, *E. fergusonii*. Error bars ± SEM. *p < 0.05; **p < 0.01; ***p < 0.001; one‐way ANOVA with Tukey's multiple comparison test. Each dot represents an individual mouse.

Intestinal epithelial barrier dysfunction has been demonstrated to be involved in the initial pathogenesis of CD.^[^ ^32^ ^]^ Given that *K. variicola* could translocate from the lumen into MAT, we intended to determine whether *K. variicola* plays a role in the disruption of the epithelial barrier. For this, we gavaged *K. variicola* (10^9^ CFU/mouse) daily into SPF mice treated with a 5‐day antibiotic cocktail for seven consecutive days. Mice were given drinking water instead of 3% DSS to exclude damage from medicinal chemicals (**Figure** **4**A). The integrity of the intestinal epithelial barrier was investigated by examining *zonula* *occludens‐1* (*ZO‐1)*, *Occludin*, *Claudin1*, and *Synaptopodin* (*SYNPO*) expression levels. As shown in Figure 4B, the mRNA expression of intestinal *ZO‐1* was significantly suppressed in *K. variicola*‐treated mice compared to those treated with *E. fergusonii* or PBS, but not for the expression of *Occludin* and *Claudin1* and *SYNPO*. Immunofluorescence staining confirmed the downregulation of ZO‐1 in mouse colonic tissues (Figure 4C,D). Electron microscopy (EM) confirmed the impairment of tight junctions in *K. variicola*‐treated mice (Figure 4E). Consistent results were observed in IL‐10^−/−^mice. *ZO‐1* expression was significantly reduced under the treatment of *K. variicola*, as demonstrated by RT‐qPCR and immunofluorescence staining (Figure 4F–H). Taken together, our results showed that *K. variicola* disrupted gut barrier integrity by inhibiting ZO‐1 expression.

### 2.5 Active T6SS in *K. variicola* Influences the Expression of ZO‐1

**Figure 5:**
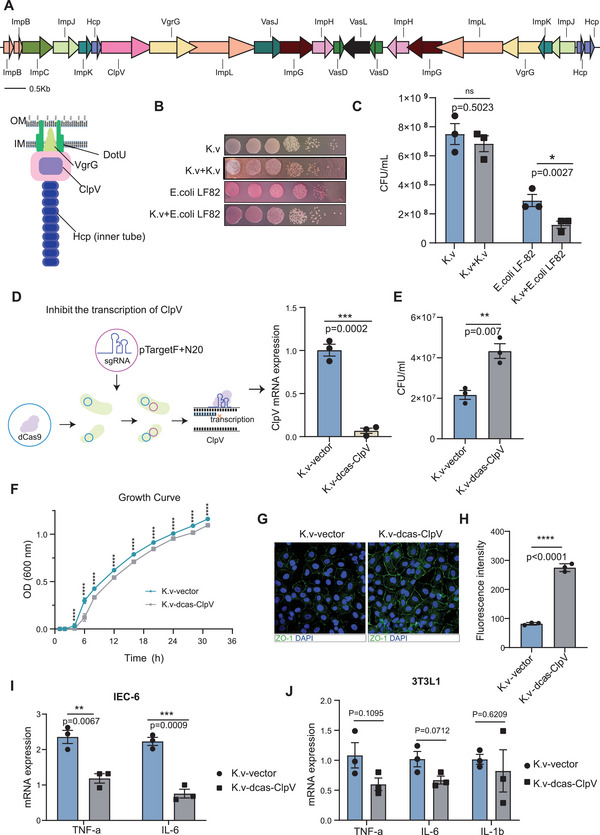
Active T6SS in *K. variicola* influences the expression of ZO‐1. A) Components of T6SS in *K. variicola* ZSLY01 based on data derived from whole‐genome sequencing. B,C) Contact inhibition of *K. variicola* ZSLY01 (white colony) toward *E. coli* LF82 (red colony). D) Schematic diagram illustrating the knockdown of ClpV by CRISPR interference (CRISPRi) system, and the mRNA expression of ClpV is significantly inhibited in K.v‐dcas‐ClpV compared to K.v‐vector. E) The contact inhibition effect toward *E. coli* LF82 was significantly attenuated in K.v‐dcas‐ClpV compared to K.v‐vector. F) The growth rate curves of K.v‐dcas‐ClpV and K.v‐vector. G,H) ZO‐1 immunofluorescent staining and quantification of K.v‐dcas‐ClpV and K.v‐vector treated IEC‐6. I,J) The pro‐inflammatory effects of K.v‐dcas‐ClpV and K.v‐vector toward IEC‐6 and 3T3L1. Bacteria‐cell co‐culture assay: MOI = 20, treated for 4 h. K.v‐vector: *K. variicola* ZSLY01 transformed with empty plasmid; K.v‐dcas‐ClpV, *K. variicola* ZSLY01 transformated with sgRNA containing plasmid. Error bars ± SEM. *p < 0.05; **p < 0.01; ***p < 0.001; ****p < 0.0001; Mann–Whitney U‐test. Results are representative of three independent experiments.

Bacterial pathobionts use various mechanisms to invade mammalian hosts, and one important strategy of gram‐negative bacteria is the release of virulence effectors through the secretion system to reach the target.^[^ ^33^ ^]^ T6SS has been demonstrated to be involved in disrupting the actin cytoskeleton.^[^ ^34^ ^]^ Moreover, stabilization of the tight junction solute barrier by ZO‐1 via coupling to the perijunctional cytoskeleton has been reported.^[^ ^35^ ^]^ Therefore, we reasoned that the disruption of the intestinal barrier by *K. variicola* may be mediated by T6SS. To test this, we first blasted the bacterial genome, which identified the existence of core components of T6SS in the genome of *K. variicola* (**Figure** **5**A). Additionally, a contact‐dependent neighbor‐killing assay was designed to assess T6SS activity in *K. variicola*. *E. coli* LF‐82 has been reported associated with ileac Crohn's disease,^[^ ^36^ ^]^ and is a lactose fermenter that grows in pink colonies on MacConkey's agar and was used as a prey cell in the inter‐bacterial killing assay. The results showed that co‐incubation of *E. coli* LF‐82 with *K. variicola* led to a remarkable reduction in the viability of *E. coli* LF‐82, indicating that active T6SS but not pseudogenes existed in *K. variicola* (Figure 5B,C).

To investigate the active role of T6SS in inducing intestinal permeability in *K. variicola*, we used a CRISPR interference (CRISPRi) system to knockdown ClpV, a core gene in T6SS (Figure 5D). ClpV is an ATPase associated with various cellular activities that functionally provide energy for sheath disassembly in T6SS. We found that ClpV inhibition in *K. variicola* led to a relatively lower growth rate, an attenuated extent of contact inhibition, and a reduced inhibition of ZO‐1 expression *K. variicola‐*dcas‐ClpV in comparison with *K. variicola‐*vector (Figure 5E–H). Besides, the in vitro pro‐inflammatory effect of *K. variicola* was also alleviated in *K. variicola‐*dcas‐ClpV‐treated IEC‐6 cells but not in 3T3L1 cells (Figure 5I,J), indicating that T6SS of *K. variicola* mainly targets the gut rather than MAT. This is consistent with our previous observation that *K. variicola* can impair the gut barrier function. Collectively, these results show that active T6SS existed in *K. variicola*, which mediated the pro‐inflammatory and barrier disruption role of *K. variicola*.

### 2.6 Dysfunction of T6SS Alleviates Colitis in Mice

**Figure 6:**
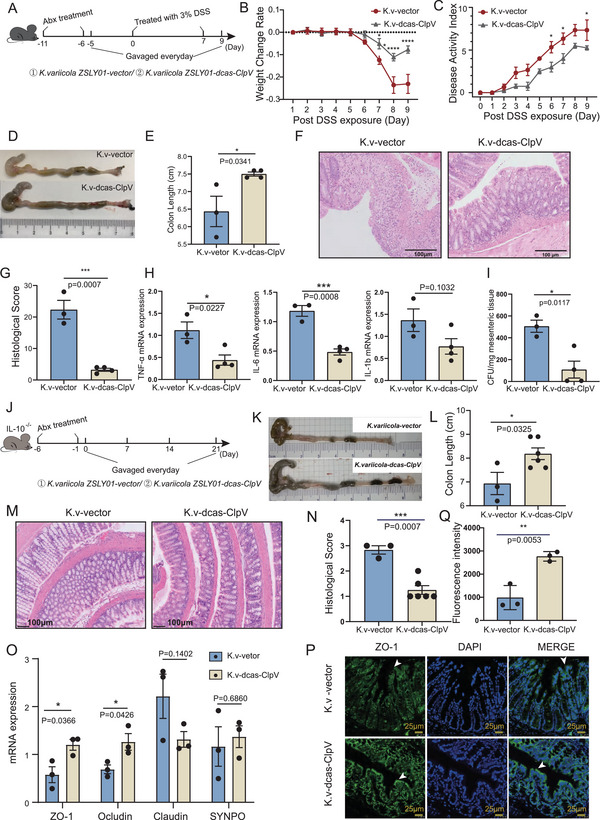
Dysfunction of type VI secretion system (T6SS) alleviates colitis in mice. A) SPF C57BL/6 male mice (*n* = 3–4) were treated with an antibiotic cocktail for 5 days. Daily bacteria (*K. variicola* ZSLY01‐vector and *K. variicola* ZSLY01‐dcas‐ClpV) continued for 14 days. To induce colitis, 3% DSS instead of drinking water was given to mice from day 0 to day 7. B) Weight loss rate and disease activity index (DAI) C) after 3% DSS treatment. D) Representative colons, E) colon length, F) representative colonic histological images (scale bar = 100 µm), G) colonic histological score of mice treated by *K. variicola* ZSLY01‐vector and *K. variicola* ZSLY01‐dcas‐ClpV. H) Expression of pro‐inflammatory cytokines (*TNF‐a*, *IL‐6*, and *IL‐1β*) in the intestine of mice. I) Detection of translocated *K. variicola* ZSLY01‐vector and *K. variicola* ZSLY01‐dcas‐ClpV in mice MAT. J) mice experiment design: SPF IL10^−/−^ mice (*n* = 3–6) were treated with an antibiotic cocktail for 5 days, and daily bacteria (*K. variicola* ZSLY01‐vector and *K. variicola* ZSLY01‐dcas‐ClpV, 1×10^9^ CFU/mouse) or PBS gavage started on day 0 and continued until euthanized on day 21. K) Representative colons, L) colon length, M) representative colonic histological images (scale bar = 100 µm), and N) colonic histological score of IL10^−/−^ mice treated by *K. variicola* ZSLY01‐vector and *K. variicola* ZSLY01‐dcas‐ClpV. O) mRNA Expression of intestinal barrier related molecules of *K. variicola* ZSLY01‐vector and *K. variicola* ZSLY01‐dcas‐ClpV treated IL10^−/−^ mice colon tissue. P,Q) ZO‐1 immunofluorescent staining and quantification of *K. variicola* ZSLY01‐vector and *K. variicola* ZSLY01‐dcas‐ClpV treated IL10^−/−^ mice colon tissue sections. K.v‐vector, *K. variicola* ZSLY01 transformated with empty plasmid; *K.v‐*dcas‐ClpV, *K. variicola* ZSLY01 transformated with sgRNA containing plasmid. Data from three independent experiments. Error bars ± SEM. *p < 0.05; **p < 0.01; ***p < 0.001; Mann–Whitney U‐test.

To address whether the dysfunction of T6SS in *K. variicola* influences its colitis‐promoting role, we carried out an in vivo mouse experiment (**Figure** **6**A). A consistent down‐regulated expression of *ClpV* in *K. variicola* was confirmed in SPF C57BL/6 mice (Figure S4A, Supporting Information). Dysfunction of T6SS did not affect the colonization of *K. variicola* in mice (Figure S4B, Supporting Information), but attenuated the colitis‐promoting effect of *K. variicola*. Mice colonized with *K. variicola*‐dcas‐ClpV showed milder colitis, as indicated by a lower weight loss rate (Figure 6B), less intensive DAI score (Figure 6C), reduced colon shortening (Figure 6D,E). Histological assessment of colonic inflammation was illustrated by decreased mucosal erosion, crypt destruction, and inflammatory cell infiltration in mice treated with *K. variicola*‐dcas‐ClpV compared to control group mice (Figure 6F,G). Additionally, a significantly decreased expression of intestinal inflammatory cytokines (TNF‐*α*and IL‐6) (Figure 6H) and *K. variicola* load in mice‐MAT was observed in mice treated with *K. variicola*‐dcas‐ClpV, compared to *K. variicola*‐vector treated controls (Figure 6I). Moreover, in line with the observations from the DSS‐induced mouse model, reduced colon inflammation was observed in *K. variicola*‐dcas‐ClpV infected SPF IL‐10^−/−^ mice compared to *K. variicola*‐vector controls (Figure 6J–N). This is consistent with a highly expressed ClpV in the fecal microbiome of patients with CD (from a publicly available database (PRJEB15371)) (Figure S5, Supporting Infromation). Additionally, dysfunction of T6SS in *K. variicola* also alleviated its inhibitory effect on ZO‐1 expression (Figure 6O,P). In the meantime, we also examined 6 of the representative genes of T6SS (Tables S6,S7, Supporting Information) in *K. variicola* ZSLY02, *K. variicola* ZSLY03, and *K. variicola* ZSLY04, and found that T6SS also existed in these 3 strains (Figure S5, Supporting Information), suggesting that all the 4 isolated strains might exacerbate colitis in mice. Taken together, these observations demonstrated that T6SS in *K. variicola* plays a vital role in disrupting intestinal permeability and triggering colitis in mice.

## 3 Discussion

In this study, we demonstrated the existence of colitogenic pathobionts in the MAT of patients with CD and explained their detrimental role in promoting intestinal inflammation. We showed that members of Enterobacteriaceae were significantly enriched in CD‐MAT. A newly defined intestinal pathogen in Enterobacteriaceae—*K. variicola* was exclusively isolated from CD‐MAT, which exacerbated colitis in mice through the disruption of the intestinal barrier by T6SS. Moreover, a higher abundance *K. variicola* was also observed in the gut microbiome of CD patients compared to healthy controls, suggesting that *K. variicola* may play an active role in CD pathogenesis.

The existence of bacterial translocation in MATs has been previously identified, including in DSS‐treated colitis in mice,^[^ ^37^ ^]^ obesity and type 2 diabetes,^[^ ^38^ ^]^ and IBD patients.^[^ ^9^ ^]^ Of them, Ha et al. (2020) first conducted the functional analysis of the translocated bacteria, which showed that members of *Clostridium* in CD‐MAT could promote the restructuring of MAT and the formation of “creeping fat.”^[^ ^9^ ^]^ Considering that extended mesenteric excision is associated with reduced surgical recurrence, we speculated that pathogenic bacteria might be involved in the pathogenesis of CD in CD‐MAT. Our previous study highlighted that Proteobacterium plylum is correlated with CD recurrence and discovered that *Achromobacter pulmonis* isolated from CD‐MAT could exacerbate colitis in mice.^[^ ^10^ ^]^ Nevertheless, as a newly defined microbial niche, there might still be numerous undiscovered microorganisms with specific functional roles in the mesenteric tissue. An expansion of Enterobacteriaceae has been regarded as a dominant dybiosis characteristic in IBD patients,^[^ ^39^ ^]^ indicating its significant role in colitis promotion. Additionally, the migration of Enterobacteriaceae from lumen into different niches of gut (e.g., colonic mucosa or ileal mucosa) has been reported frequently.^[^ ^12^, ^13^, ^14^ ^]^ Here, we observed that Enterobacteriaceae were significantly enriched in MAT of CD patients, consistent with results from a previous study in which the mesenteric bacteria were translocated from the intestinal lumen. Additionally, we observed heterogeneity in the distribution of Enterobacteriaceae in CD‐MAT. MAT in the high‐E group had much lower bacterial diversity, indicating an intensive translocation of a specific bacterial group. Besides, pathogenic or opportunistic bacteria (i.e., *Pseudomonas*, *Burkholderia*, and *Streptococcus*) accumulated in the bacterial community from the high‐E group, suggesting that Enterobacteriaceae may play a detrimental role in the pathogenesis of CD.

Based on the culture‐omics data, we identified that *K. variicola* belonging to Enterobacteriaceae existed exclusively in CD‐MAT. *K. variicola* is a member of the *Klebsiella* genus, which was initially regarded as a plant‐associated pathogen and subsequently recovered from human clinical specimens.^[^ ^40^ ^]^ It has been suggested that approximately 20% of human isolates previously thought to be *K. pneumoniae* are *K. variicola*/KpIII or *K. quasipneumoniae*/KpI.^[^ ^41^ ^]^ In the current study, through whole‐genome sequencing and genotyping‐based phylogenic analysis, we were able to differentiate *K. variicola* from the other three species. Genes associated with adhesion, invasion, and motility were observed in the *K. variicola* genome, indicating its translocation ability. Additionally, the genomic data of *K. variicola* displayed a wide metabolic versatility (including lipid metabolism) and anti‐oxidative damage ability, which may help enable *K. variicola* to successfully survive in the MAT niche. Additionally, although not clearly explained by Ha et al. (2020), *Klebsiella* spp. was also exclusively isolated from the MAT of patients with IBD, confirming that *Klebsiella* spp. commonly co‐existed with mesentery tissue.^[^ ^9^ ^]^

Previously, *K. pneumoniae* and *K. oxytoca* in *Klebsiella* spp. have been reported to have colitogenic roles in the initiation and perpetuation of intestinal inflammation.^[^ ^25^, ^26^, ^27^ ^]^ However, whether *K. variicola* has a colitogenic role remains unknown. Here, we isolated 4 strains of *K. variicola* from 3 different CD‐MAT samples and observed that they all could induce a significant pro‐inflammatory phenotype upon cell‐bacterial co‐culture in vitro. Furthermore, we confirmed that *K. variicola* exacerbates colitis in mice. To our knowledge, this is the first evidence that colitogenic *K. variicola* exists in the mesenteric tissue. Previous studies have shown that mice deficient in Muc2 have increased translocation of both commensal and pathogenic bacteria, supporting that epithelial barrier dysfunction can accelerate passive migration.^[^ ^42^ ^]^ Considering *K. variicola* is a colitogenic pathogen translocated from the intestinal lumen, we propose that *K. variicola* may promote intestinal barrier dysfunction. As expected, we observed that *K. variicola* promoted intestinal barrier disruption in a mouse model without DSS treatment. Bacterial toxins or effectors produced by the bacterial secretion system contribute to the intestinal barrier disruption. For example, the zonula occludens toxin, a toxin produced by *Vibrio cholerae*, interacts with the extracellular domains of tight junction proteins (occludins, ZO‐1) to disrupt epithelial integrity.^[^ ^43^ ^]^ Actin cytoskeleton regulate and stabilize TJs and AJs. *C. rodentium* uses type III secretion systems to reorganize the cytoskeleton and consequently alter the architecture and integrity of tight junction proteins.^[^ ^44^ ^]^ T6SS in *V. cholerae* can induce actin cross‐linking, which is associated with intestinal inflammation.^[^ ^45^ ^]^ In this study, using whole‐genome sequencing, we identified the presence of gene clusters encoding core T6SS components in *K. variicola*. Meanwhile, the existence of representative genes of T6SS were also identified in the other 3 stains of *K. variicola*. ClpV ATPase is crucial for T6SS functioning, and the inhibition of ClpV significantly reduced the *K. variicola* induced inflammatory effect in vitro and in vivo and alleviated its inhibitory effect on ZO‐1 expression. Importantly, we validated that *K. variicola* and ClpV were enriched in the gut microbiome of patients with CD, suggesting that pathogenic bacteria with barrier disruption role as well as T6SS are closely related to the pathogenesis of CD.

However, there were limitations to this study. We proposed that *K. variicola* is translocated from the intestinal lumen, but the fecal samples paired with MAT specimens are not available. The existence of higher level of *K. variicola* was thus validated by using published data (PRJEB15371). Additionally, we identified that T6SS plays an active role in promoting inflammation; however, the function of the specific core protein in T6SS remains to be demonstrated.

In conclusion, this study provides a framework to show that colitogenic pathobionts exist in the CD mesentery, which can induce a breakdown of the epithelial barrier and the release of pro‐inflammatory cytokines (Figure S6, Supporting Information), thereby contributing to disease progression. Targeting microbial‐derived activities may help in the development of treatments for CD progression.

## 4 Experimental Section

### Sample Collection

MATs were collected by surgical resection of 48 patients with CD and 16 non‐CD controls (patient diagnosis of CRC). The diagnosis of CD was based on an integrated observation of endoscopy, clinical, and histopathological data. Clinical information of the recruited cohort is listed in Table S1, Supporting Information. The diagnosis of CRC was made according to clinical guidelines and typical histopathological results. Exclusion criteria included patients unwilling to provide informed consent, took antibiotics within two weeks, being contraindicated to surgery, had acute gastrointestinal infection, being pregnant, had known bleeding disorder, or diagnosed with end‐stage malignancy. The ethics committee of the Sixth Affiliated Hospital of Sun Yat‐sen University (Guangzhou, CN) approved the protocol (No. 2020ZSLYEC‐012). Written informed consent was obtained from all participants. To prevent spill‐over contamination during surgery, MATs were sampled before opening the intestine. After resection, the specimens were transferred immediately into 50 mL sterile collection tubes (Corning) and transported to the central laboratory of the authors’ hospital on ice within 30 min of resection. Upon arrival, the specimens were rinsed in sterile PBS to remove the surface blood, which was subsequently subsampled into 4–5 pieces in an anaerobic chamber. MAT from non‐CD controls was resected from the terminal ileum at the same location from where the CD‐MAT was collected.

### Bacterial Strains and Culture Conditions

The strains isolated from MATs are listed in Table S3, Supporting Information. Of these, *Klebsiella variicola* was identified as a CD‐specific species for functional and mechanistic analysis in the current study. In total, 4 strains of *K. variicola* were isolated from 3 different CD‐MAT samples, which were named as *K. variicola* ZSLY01, *K. variicola* ZSLY02, *K. variicola* ZSLY03, and *K. variicola* ZSLY04. All of them showed pro‐inflammatory ability toward the intestinal cell line, IEC‐6 (detected by cell‐bacteria co‐culture experiment). *K. variicola* ZSLY01 was chosen as representative strain for the subsequent in vivo and in vitro experiments. In the following context, all *K. variicola* mentioned refers to *K. variicola* ZSLY01 unless otherwise specified. Additionally, the following reference strains were used in this study: reference adherent/invasive *Escherichia coli* strain LF82, *E. coli* DH5*α* and a commensal strain of *Escherichia fergusonii* isolated from SPF C57BL/6 mice. *Escherichia coli* strain LF82 had been reported associated with ileac Crohn's disease, which is a lactose fermenter that grows in pink colonies on MacConkey's agar and was used as a prey cell in the inter‐bacterial killing assay; while the latter two were used as a non‐pathogenic bacterial control in the authors’ cell and mouse experiments. These bacteria were grown in brain‐heart infusion (BHI) medium under aerobic conditions at 37 °C for 14–16 h.

### DNA Extraction, 16S rRNA Gene Sequencing, and Data Analysis

DNA was extracted from MAT samples using the bead‐beating method with a DNeasy Blood and Tissue Kit (QIAGEN, MD). The hypervariable V4 region of the 16S rRNA gene was amplified by PCR using dual barcode primers as previously described.^[^ ^46^ ^]^ Mixtures with denatured amplicons and 20% PhiX Control v.3 were then sequenced on Hiseq 2500 (Illumina, 2×250 bp paired‐end reads). Quantitative Insights into Microbial Ecology 2 (QIIME II) using reference parameters was used for demultiplexing and quality filter.

### Localization of Bacteria by Fluorescence In Situ Hybridization (FISH)

Paraffin‐embedded MAT histopathological sections were de‐waxed and hydrated first, followed by the overnight hybridization step performed at 46 °C. EUB338 probe (5′‐GCTGCCTCCCGTAGGAGT‐3′, with Cy3 label) and specific probe designed for *Klebsiella* spp. (5′‐CCTACACACCAGCGTGCCA‐3′, with FITC label) were diluted to a final concentration of 5 ng µL^−1^ in the hybridization buffer (20 mm Tris‐HCl, pH 7.4, 0.9 m NaCl, 0.1% SDS, and 35% formamide). After hybridization, the sections were rinsed with wash buffer (20 mm Tris‐HCl, pH 7.4, and 0.9 m NaCl) for three times (5 min/time), with Tris‐buffer for three times (3 min/time) at 48 °C and 1 min in ddH_2_O. Dry the slides in an oven at 46 °C for 10 min, and then mounted the slides by Prolong anti‐fade mounting media with DAPI (Life Technologies). Microscopic observations were performed by Leica Laser Scanning confocal microscope (Leica TCS‐SP8, Leica Microsystems Inc, Buffalo Grove, IL, USA).

### Bacterial Cell Co‐Culture

Murine preadipocyte 3T3L1 or rat intestinal epithelial cell line IEC‐6 were co‐cultured with bacteria (*K. variicola* ZSLY01, *K. variicola* ZSLY02, *K. variicola* ZSLY03, *K. variicola* ZSLY04, *E. fergusonii*, *K. variicola*‐vector, or *K. variicola*‐dcas‐ClpV) at a multiplicity of infection (MOI) of 20 at 37 °C, 5% CO_2_ for 4 h. Then, they were washed with warm PBS containing antibiotics (kanamycin and ampicillin at 100 ng mL^−1^ for strains of *K. variicola* and *E. fergusonii* treated groups; kanamycin and spectinomycin at 500 ng mL^−1^ for *K. variicola*‐vector or *K. variicol*a‐dcas‐ClpV treated groups). Cells were then cultured for another 1 h in DMEM (Gibco) containing the antibiotics mentioned above. RNA from cells was extracted using TRIzol reagent (Thermo Fisher Scientific) and the gene expression was measured by qPCR.

### Genome Sequencing, Assembly, and Annotations of *K. variicola*

The whole genome sequencing of *K. variicola* ZSLY01 were carried out on the Nanopore sequencing platform by Biomarker Technologies Corporation (Beijing, China). To assemble the filtered data, the Canu v1.5 package was used.^[^ ^47^ ^]^ Racon v3.4.3 and Circlator v 1.5.5 were employed to check the quality of the assembly.^[^ ^48^ ^]^ Gene prediction was carried out using the Prodigal (v2.63) software program.^[^ ^49^ ^]^ RepeatMasker (v4.0.5) was used to predict repetitive sequence.^[^^][50]^ PhiSpy v2.3 and CRT v1.2 were used for prophage prediction and CRISPR identification, respectively.^[^ ^51^, ^52^ ^]^ Functional characterization of the identified genes was completed by diamond with the E‐value threshold of 1E‐5 against the databases of the NCBI Non‐Redundant protein database (NR), Swiss‐Prot, Clusters of Orthologous Groups (COG), Kyoto Encyclopedia of Genes and Genomes (KEGG), and Gene Ontology (GO).

### Mice Experiment

C57BL/6 and IL‐10^−/−^ mice (6–8 weeks old) were purchased from Gem Pharmatech Co. Ltd. (Guangzhou) and Beijing HFK Bioscience Co. Ltd. (Beijing) and were housed under a 12‐h light‐dark cycle in specific pathogen‐free (SPF) facilities. All animal study was performed in accordance with guidelines approved by the ethics committee of the South China Agricultural University (No. 2019c026). To explore the pro‐inflammatory role of *K. variicola* in the pathogenesis of colonic inflammation, the dextran sulfate sodium (DSS)‐induced colitis model and spontaneously developing colitis model (IL‐10^−/−^ mice) were used.^[^ ^40^ ^]^ For DSS‐induced colitis model, male C57BL/6 mice were daily gavaged with bacteria (1×10^9^ CFU/mouse) for 14 days after being treated with the antibiotic cocktail (ampicillin 0.2 g L^−1^, metronidazole 0.2 g L^−1^, Neomycin 0.2 g L^−1^, vancomycin 0.1 g L^−1^) in the drinking water for 5 days. 3% DSS was administered on day 0, which lasted for seven consecutive days, and then changed by regular water for two days. The mice were monitored for weight loss, stool consistency, and hemoccult during the experiments, and the disease activity index (DAI) was calculated from the observations above. For spontaneously developing colitis model, IL‐10^−/−^ mice were treated with the above antibiotic cocktail in the drinking water for 5 days, followed by daily gavage of bacteria (1×10^9^ CFU/mouse) for 21 consecutive days. During the experiment, mice were monitored for weight loss, stool consistency, and hemoccult, and the DAI was calculated from the observations above. In addition, to explore the impact of *K. variicola* on intestinal barrier function, male SPF C57BL/6 mice were gavaged with *K. variicola* for 7 consecutive days after a 5‐day‐antibiotic cocktail treatment.

### Contact‐Dependent Neighbor‐Killing Assay

The contact‐dependent neighbor‐killing assay was performed according to a previously established protocol, with minor modifications.^[^ ^53^ ^]^ Briefly, *E. coli* LF82 and *K. variicola* (or *K. variicola‐*vector, *K. variicola‐*dcas‐ClpV), which were defined as prey and predator cells, respectively, were harvested after overnight growth, washed, and concentrated to (OD_600_) in PBS. Predator cells were well mixed with *E. coli* at a ratio of 1:1, which were then spotted onto pre‐warmed LB agar plates. The plates were incubated at 37 °C for 4 h and then each spot was cut out in the LB plate and suspended in sterile PBS. The number of prey cells recovered on the MAC agar plate was counted to determine the colony‐forming units per mL.

### ClpV Knockdown by CRISPR/dCas9‐Assisted System

The CRISPR interference (CRISPR i) system was used to inhibit ClpV expression in T6SS. This system worked from two plasmids: one plasmid encoding constitutive dCas9 (Addgene, plasmid #65006), and another encoding constitutively expressed sgRNA targets (pTargetF, Addgene, plasmid #62226). The newly designed sgRNA on the website (http://www.rgenome.net/cas‐designer/) targeting the gene *ClpV* was cloned into plasmid #62226 with a single *SpeI* restriction site. Construction of the complete N20 containing plasmid was conducted by single digestion with *SpeI*, template excision with *DpnI*, and fragment ligation with T4 DNA ligase (Thermo Scientific). This plasmid was subsequently transformed into *E. coli* DH5*α* and subjected to Sanger sequencing to confirm successful insertion (RuiBiotech). To create *K. variicola‐*dcas‐ClpV, competent *K. variicola* cells were first transformed with dcas9 with kanamycin resistance and then prepared for competent cells for the second transformation with N20‐pTargetF with spectinomycin resistance. The original pTargetF vector without N20 was transformed into *K. variicola‐*dcas competent cells to obtain the *K. variicola‐*vector as a negative control. The inhibition efficiency of ClpV was evaluated by qPCR.

### Statistical Analysis

GraphPad Prism 8 was used for statistical analyses, except for ROC, which was analyzed using an online bioinformatics analysis platform (https://www.omicstudio.cn/tool). Significance between two groups was determined by an unpaired two‐tailed Student's *t*‐test. Significance between data that did not pass the normal distribution was determined with the unpaired two‐tailed Mann–Whitney test. For comparisons between multiple groups, an ordinary one‐way or two‐way analysis of variance (ANOVA) was utilized, followed by Tukey's and Dunnett's tests. For comparison between multiple groups with two factors, two‐way ANOVA was used, followed by Tukey's multiple comparison test. The specific statistical details were shown in the figure captions and the source data. Differences were regarded as significant when *p* < 0.05. Unless otherwise indicated, data for all experiments are presented as the mean ± standard error of the mean (SEM).

## Conflict of Interest

The authors declare no conflict of interest.

## Author Contributions

J.G., J.Y., and S.Y. contributed equally to this work. Z.H., J.G., and P.L. supervised the study and designed the research. J.G., J.Y., S.Y., J.W., C.L., Z.L., and W.M.C. performed the experiments. J.G., J.Y., and S.Y. prepared the manuscript (J.G drafted the initial manuscript). J.Y. and J.K. helped with sample collection. Y.X. helped with clinical data collection. J.W. and Y.C. helped with data analysis. All authors read, revised, and approved the final manuscript.

## Supporting information

Supporting Information

## Data Availability

The data that support the findings of this study are available from the corresponding author upon reasonable request.
