# Supplementary material for: Mesenteric Adipose Tissue‐Derived Klebsiella variicola Disrupts Intestinal Barrier and Promotes Colitis by Type VI Secretion System
Source: Adv Sci (Weinh). 2023 Feb 19;10(12):2205272. doi: 10.1002/advs.202205272 (PMC10131791; doi:10.1002/advs.202205272)
Supplement: Supplementary file 1 — Supporting Information [file ADVS-10-2205272-s001.pdf]

ADVANCED SCIENCE
Open Access

# Supporting Information

for *Adv. Sci.*, DOI 10.1002/advs.202205272

Mesenteric Adipose Tissue-Derived *Klebsiella variicola* Disrupts Intestinal Barrier and Promotes Colitis by Type VI Secretion System

*Junli Gong, Jing Yu, Shengmei Yin, Jia Ke, Jinjie Wu, Chen Liu, Zhanhao Luo, Wai Ming Cheng, Yaozu Xie, Yuan Chen, Zhen He* and Ping Lan**

## Supporting Information

### Experimental Procedures

*MAT bacteria cultivation*: MAT (approximately 100 mg) was homogenized using Sample Freezing Grinder (Lu Ka, LUKYM-I) in 1 mL of sterile PBS. The samples were serially diluted and spread onto agar media, including brain heart infusion media (BHI, BD), BHI supplemented with vitamin K (0.5 mg/L), heme (5 mg/L), MacConkey agar (MAC, Oxoid), and tryptic soy agar (TSA, BD). The plates were incubated under aerobic conditions for 14-16 h or under anaerobic conditions at 37°C for up to 96 hours. Distinct bacterial colonies were selected and re-streaked for purification. The nearly full-length 16S rRNA gene was amplified using the 27F/1492R primer set for taxonomical assignment **(Table S2)**. Amplicons were submitted to Rui Biotech (Guangzhou, CN) for Sanger sequencing. Each colony was identified by sequencing using the Basic Local Alignment Search Tool available on NCBI. The SILVA database was used for taxonomic hierarchy information reference.

*DNA extraction, 16S rRNA gene sequencing and data analysis*: DNA was extracted from MAT samples using the bead-beating method with a DNeasy Blood & Tissue Kit (QIAGEN, MD). The hypervariable V4 region of the 16S rRNA gene was amplified by PCR using dual barcode primers as previously described [46]. Briefly, amplicons were purified by AMPure XP (Beckman Coulter), and quantified with Quant-iT PicoGreen ds DNA Assay Kit (Thermo Fisher Scientific). Equal amounts of each amplified DNA were pooled together, which were subsequently qualified and quantified by Bioanalyzer 2100 using the High sensitivity DNA kit (Agilent) as well as the KAPA Library Quantification Kit for Illuminan (Kapa Biosystem). Mixtures

with denatured amplicons and 20% PhiX Control v.3 were then sequenced on Hiseq 2500 (Illumina, 2×250 bp paired-end reads). Quantitative Insights into Microbial Ecology 2 (QIIME II) using reference parameters was used for demultiplexing and quality filter. Sequences having 100% similarity were clustered into amplicon sequence variant (ASV). 16S rRNA gene sequence variants were aligned to the Greengenes database (http://greengenes.secondgenome.com) for taxonomic assignment. The relative abundance of each ASV was calculated from the proportion of the sum of sequences in each sample.

*Bacteria invasion assay*: Murine preadipocyte 3T3L1 cells were seeded in 24-well cell culture plates at a density of $1\times10^5$ cells/well. Bacterial cells (*K. variicola* and *E. coli* DH5α) were washed three times with PBS and re-suspended in DMEM without antibiotics. Bacteria were added to the cell plates at a multiplicity of infection (MOI) of 20 and incubated at 37°C in 5% $CO_2$ for 4 h. Then, the in-well co-cultured systems were washed thrice with warm PBS, followed by an incubation in DMEM (10% FBS) containing antibiotics (kanamycin and ampicillin at 100 ng/mL) at 37°C, 5% $CO_2$ for another 1 h to kill extracellular bacteria. Cells were then lysed with 0.5% Triton X-100 in PBS, and internalized bacteria were enumerated by plate counting.

*Quantification of K. variicola in mice mesenteric adipose tissue and clinical sample*: To determine the abundance of *K. variicola* in the clinical samples (mesenteric adipose tissue (MAT) and the intestine tissue) as well as mice MAT, absolute quantitative PCR (qPCR) was performed. Amplification was carried out in triplicate with SYBR Green qPCR in accordance with the manufacturer's protocol (Biomarker, CN). A standard curve was plotted using serially diluted bacterial DNA extracted from a known amount of *K. variicola* (e.g., $5\times10^9$ CFU). With this standard

curve for qPCR, the abundance of *K. variicola* in each sample was calculated according to the detected CT value. Specific qPCR primers for *K. variicola* were designed based on whole genome sequencing data of *K. variicola* **(Table S2)**.

*Growth Curves*: Growth curves were monitored using an automatic biological growth reactor (RTS-1C, Unk Bio). Overnight cultures of *K. variicola*-vector and *K. variicola*-dcas-ClpV were diluted in 25 mL LB supplemented with kanamycin (50 μg/mL) and spectinomycin (50 μg/mL). Bacterial growth at $OD_{600}$ was measured every ten minutes (Rev. Spin period: five seconds, 220 rpm/min at 37 °C).

*Immunofluorescence for Zonula occludens-1 (ZO-1)*: The mouse colonic tissue sections were freshly isolated and fixed with 10% formalin before embedding in paraffin wax. The whole staining was performed on paraffin-embedded sections (4 μm). After deparaffination with dimethylbenzene and rehydration with ethanol, the slides were immersed in boiling sodium citrate buffer (10 mM, pH=6.0) for 10 min to retrieve antigen. The slides were blocked with PBS containing 0.6% Triton X-100 for 15min, and then washed in PBST (0.1% Tween 20 in PBS) 3 times, each 5 min. The tissue samples were immune-stained with primary antibodies by incubating overnight at 4°C. Following incubation, the tissue sections were rinsed with PBST 3 cycles as previously described. After that, slides were incubated with Alexa Fluor 488 conjugated secondary antibodies for 30 min at room temperature. Tissues were mounted in Prolong antifade with DAPI reagent, then slides were covered by a coverslip and sealed the edges to prevent drying. Specimens were examined with Leica Laser Scanning confocal microscope. The quantification of ZO-1 was performed with Image J.

*Transmission Electron Microscopy (TEM)*: Colon tissue (1 $mm^3$) was collected from *K. variicola* ZSLY01, *E. fergusonii*, or PBS-treated C57BL/6 mice and fixed in electron microscopy fixatives (Servicebio, CN). Ultrathin sections were prepared using a Reichert Ultracut E ultramicrotome (Leica Microsystems). Digital images were obtained using TEM (Hitachi H-7000, equipped with an AMT CCD camera XR-41, Hitachi, Japan).

**Figure Legend**

**Figure S1. Linear discriminant analysis effect size (LEfSe) analysis showing the differential distribution of microbiota in mesenteric adipose tissue (MAT) with high and low abundance of Enterobacteriaceae.** High E: high abundance of Enterobacteriaceae; Low E: low abundance of Enterobacteriaceae.

**Figure S2. Genomic analysis and the invasion as well as pro-inflammatory capacity of *K. variicola* ZSLY01.** (A) Functional analysis of genomic genes in *K. variicola* ZSLY01; (B) Genes involved in protecting against oxidative damage; (C) Invasion capacity of *K. variicola* ZSLY01 into 3T3L1 (mice preadipocytes). *E. coli* DH5α was employed as a control group. (D) The pro-inflammatory capacity of K. *variicola* ZSLY01 towards 3T3L1.

**Figure S3. *K. variicola* exacerbate colitis in SPF mice without antibiotics treatment.** (A) mice experiment design: SPF C57BL/6 male mice (n=4-5) were daily gavaged with *K. variicola* or PBS for 14 days. To induce colitis, 3% dextran sodium sulfate (DSS) instead of drinking water was given to mice from day 0 to day 7. (B) weight loss rate and (C) disease activity index (DAI) after 3% DSS treatment. (D-E) Representative colons and colon length. (F-H) Expression of pro-inflammatory cytokines (*TNF-a, IL-6, and IL-1β*) in the mice intestines. Error bars ± SEM. *P<0.05; **P<0.01; Each dot represents an individual mouse; two-way ANOVA with Tukey's multiple comparison test (B&C); one-way ANOVA with Tukey's multiple comparison test (E); unpaired two-tailed Student's t-test (F-H).

**Figure S4. Down-regulated expression of *ClpV* in *K. variicola* derived from mice and the comparable colonization ability between K.v-vetor and K.v-dcas-ClpV.** K.v-vector, *K. variicola* transformated with empty plasmid; *K.v*-dcas-ClpV, *K.variicola* transformated with sgRNA containing plasmid. Error bars±SEM. *P<0.05; **P<0.01; ***P<0.001; unpaired two-tailed Student's t-test.

**Figure S5. Detection of T6SS in the fecal microbiome of patients with CD (A) and that in the strains of *K. variicola* (B).** The sequencing data of fecal microbiome of patients with CD is from a publicly available database (PRJEB15371). K.v ZSLY 01, *K. variicola* ZSLY 01; K.v ZSLY 02, *K. variicola* ZSLY 02; K.v ZSLY 03, *K. variicola* ZSLY 03; K.v ZSLY 04, *K. variicola* ZSLY 04; *P. distasonis, Parabacteroides distasonis*

**Figure S6. Summary schema showing the proposed mechanism by which MAT-derived *K. variicola* promotes intestinal colitis**

**Figure S1**

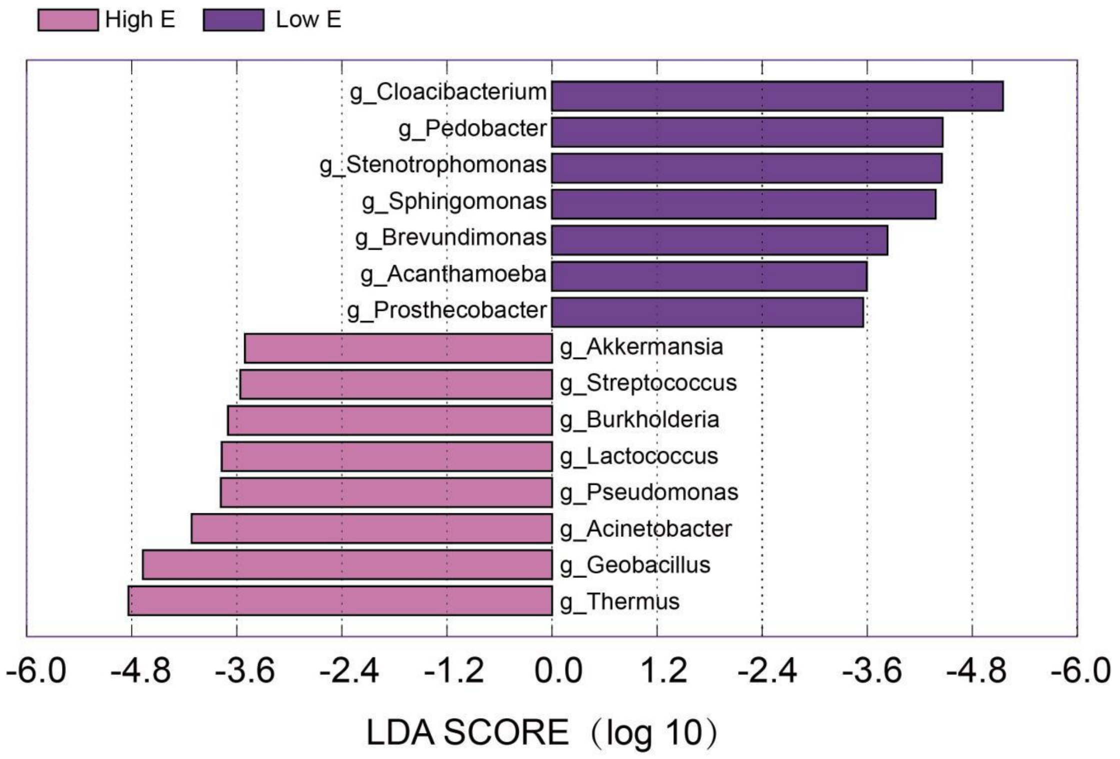
High E
Low E
g_Cloacibacterium
g_Pedobacter
g_Stenotrophomonas
g_Sphingomonas
g_Brevundimonas
g_Acanthamoeba
g_Prosthecobacter
g_Akkermansia
g_Streptococcus
g_Burkholderia
g_Lactococcus
g_Pseudomonas
g_Acinetobacter
g_Geobacillus
g_Thermus
-6.0
-4.8
-3.6
-2.4
-1.2
0.0
1.2
-2.4
-3.6
-4.8
-6.0
LDA SCORE（log 10）

**Figure S2**

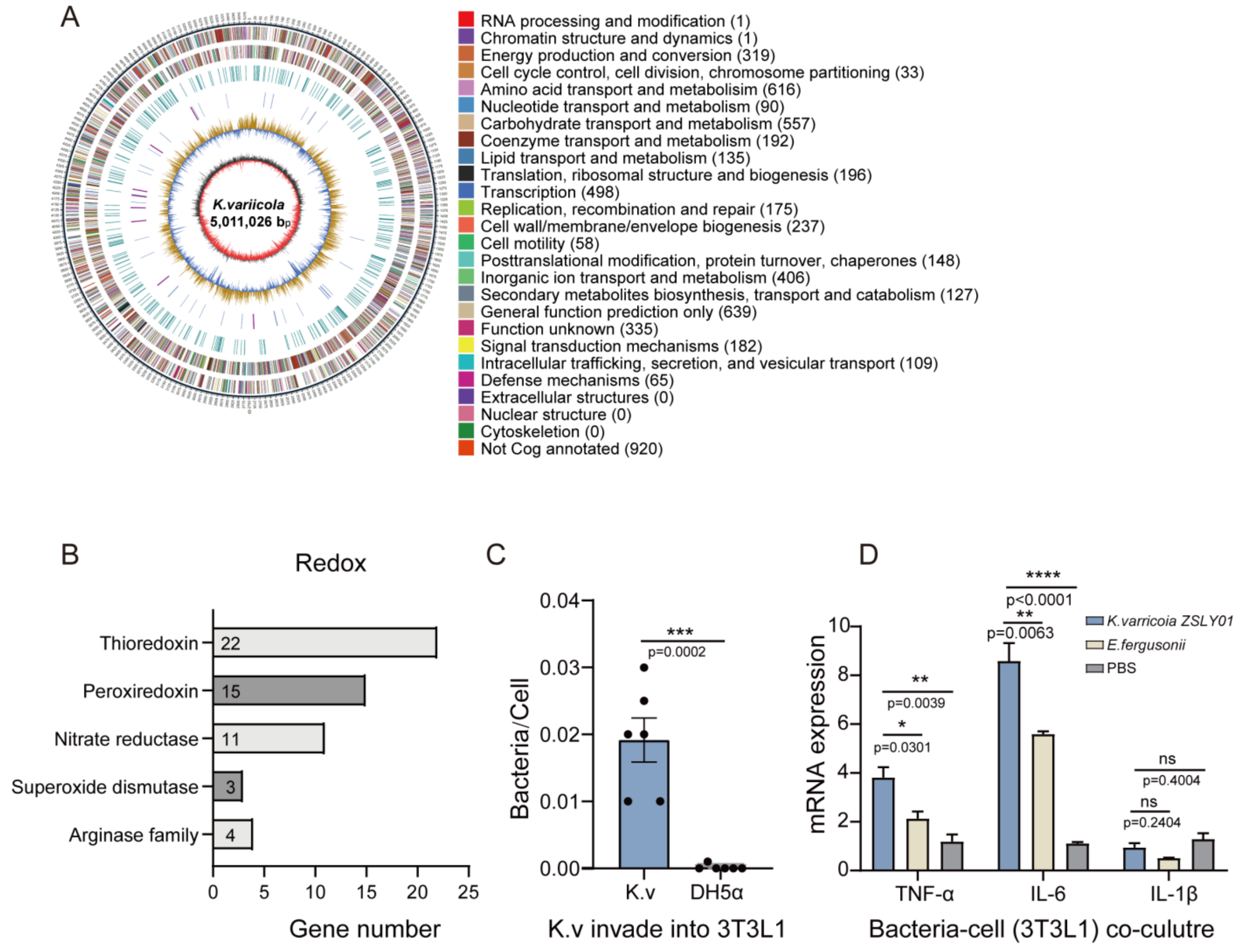

A
K.variicola
5,011,026 bp
RNA processing and modification (1)
Chromatin structure and dynamics (1)
Energy production and conversion (319)
Cell cycle control, cell division, chromosome partitioning (33)
Amino acid transport and metabolisim (616)
Nucleotide transport and metabolism (90)
Carbohydrate transport and metabolism (557)
Coenzyme transport and metabolism (192)
Lipid transport and metabolism (135)
Translation, ribosomal structure and biogenesis (196)
Transcription (498)
Replication, recombination and repair (175)
Cell wall/membrane/envelope biogenesis (237)
Cell motility (58)
Posttranslational modification, protein turnover, chaperones (148)
Inorganic ion transport and metabolism (406)
Secondary metabolites biosynthesis, transport and catabolism (127)
General function prediction only (639)
Function unknown (335)
Signal transduction mechanisms (182)
Intracellular trafficking, secretion, and vesicular transport (109)
Defense mechanisms (65)
Extracellular structures (0)
Nuclear structure (0)
Cytoskeleton (0)
Not Cog annotated (920)
B
Redox
Thioredoxin 22
Peroxiredoxin 15
Nitrate reductase 11
Superoxide dismutase 3
Arginase family 4
0 5 10 15 20 25
Gene number
C
Bacteria/Cell
0.04 0.03 0.02 0.01 0.00
***
p=0.0002
K.v
DH5α
K.v invade into 3T3L1
D
mRNA expression
10 8 6 4 2 0
K.varricoia ZSLY01
E.fergusonii
PBS
**
p=0.0039
*
p=0.0301
****
p<0.0001
**
p=0.0063
ns
p=0.4004
ns
p=0.2404
TNF-α
IL-6
IL-1β
Bacteria-cell (3T3L1) co-culutre

# Figure S3

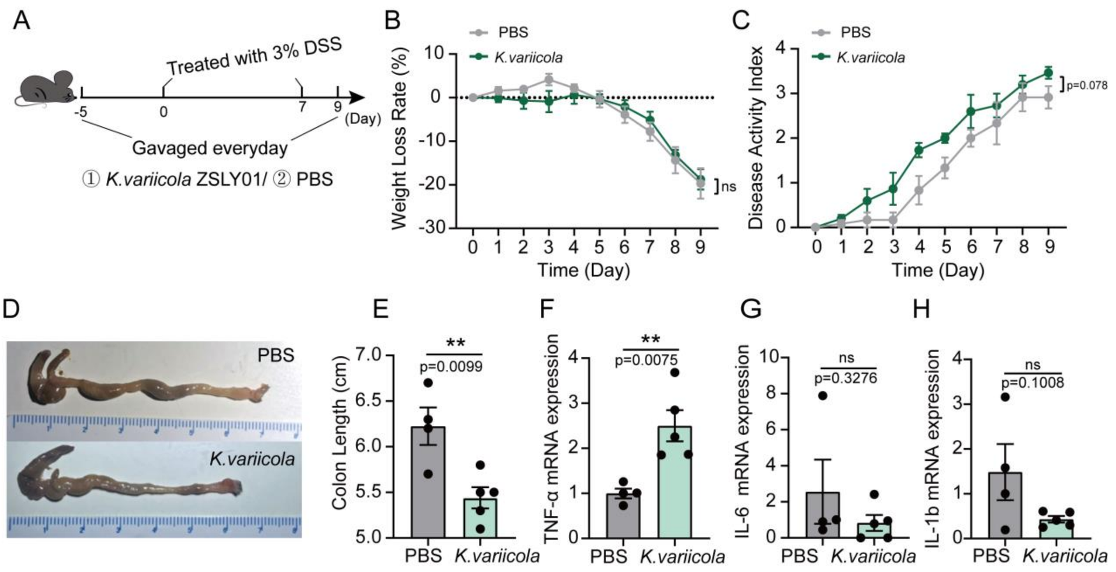
A
Treated with 3% DSS
-5
0
7
9
(Day)
Gavaged everyday
① K.variicola ZSLY01/ ② PBS
B
PBS
K.variicola
Weight Loss Rate (%)
Time (Day)
ns
C
Disease Activity Index
Time (Day)
p=0.078
D
PBS
K.variicola
E
Colon Length (cm)
**
p=0.0099
F
TNF-α mRNA expression
**
p=0.0075
G
IL-6 mRNA expression
ns
p=0.3276
H
IL-1b mRNA expression
ns
p=0.1008

**Figure S4**

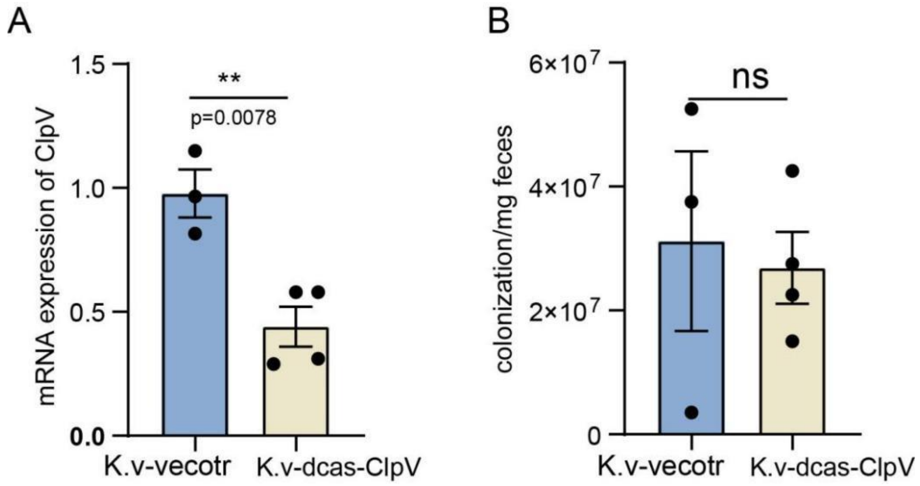
A
1.5
1.0
0.5
0.0
mRNA expression of ClpV
**
p=0.0078
K.v-vecotr
K.v-dcas-ClpV
B
6×10^7
4×10^7
2×10^7
0
colonization/mg feces
ns
K.v-vecotr
K.v-dcas-ClpV

**Figure S5**

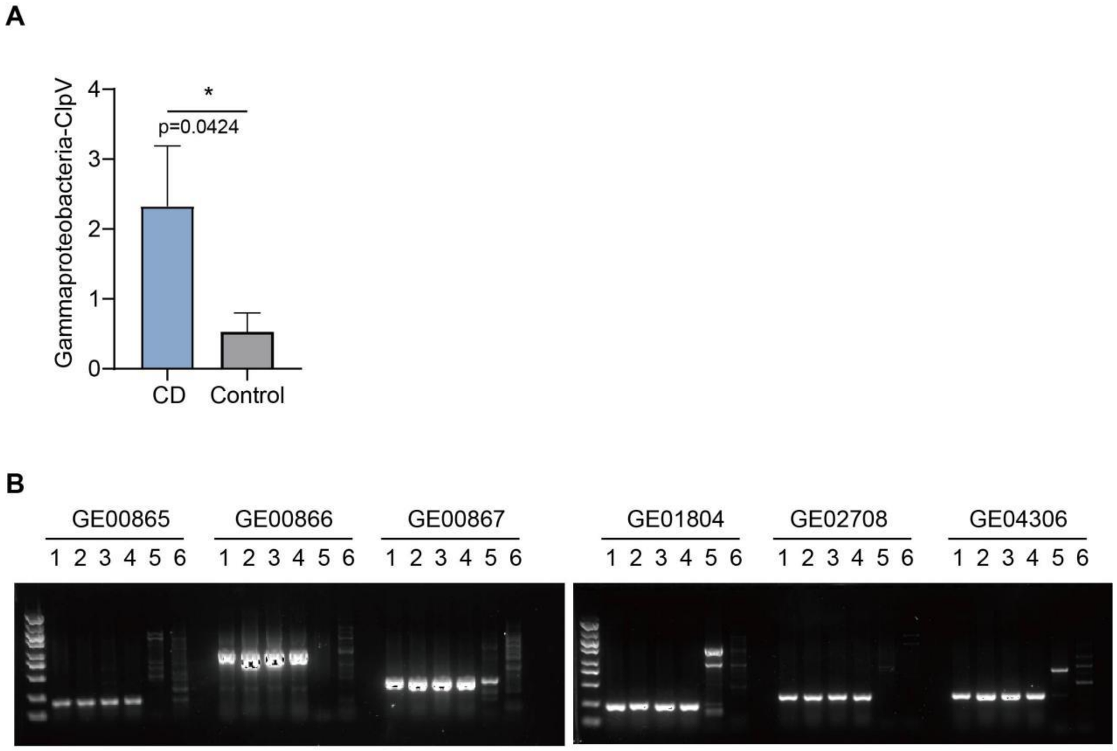


Lane 1: K.v ZSLY01
Lane 2: K.v ZSLY02
Lane 3: K.v ZSLY03
Lane 4: K.v ZSLY04
Lane 5: *P. distasonis*
Lane 6: Elution buffer

**Figure S6**

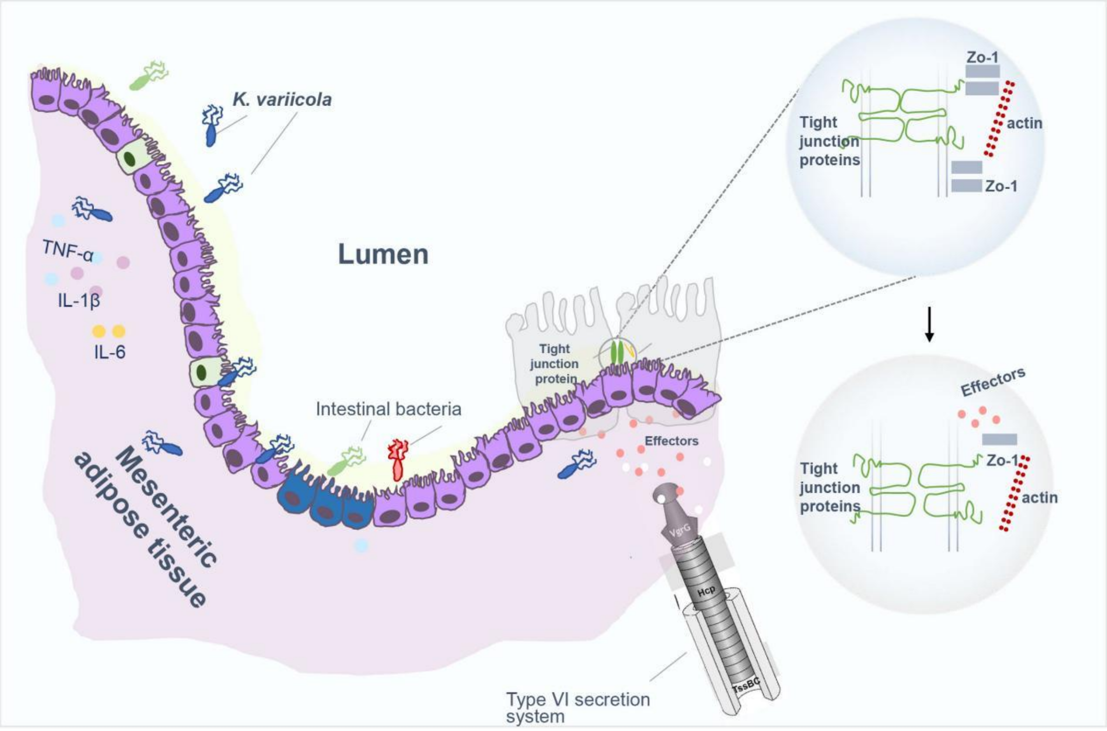
K. variicola
Lumen
TNF-α
IL-1β
IL-6
Mesenteric adipose tissue
Intestinal bacteria
Tight junction protein
Effectors
VgrG
Hcp
TssBC
Type VI secretion system
Zo-1
Tight junction proteins
actin
Zo-1
Effectors
Tight junction proteins
Zo-1
actin

Table S1

**Table S1. Clinical information of CD patients and non-CD controls involved in this study**

| | | **CD patients (n=48)** | **non-CD control (n=16)** |
|---|---|---|---|
| Gender | Male | 30 | 7 |
| | Female | 18 | 9 |
| Family history of IBD | Yes | 0 | |
| | No | 48 | |
| Median age at diagnosis (min. - max.) (year) | | 31 (11-55) | 60 (25-85) |
| Median duration of disease (min. - max.) (year) | | 7 (4-18) | |
| Active | Yes | 24 | |
| | No | 24 | |
| Limber[1] | Grade I | 1 | |
| | Grade II | 25 | |
| | Grade III | 16 | |
| | Grade IV | 6 | |
| Montreal classification[2] | A1 | 5 | |
| | A2 | 39 | |
| | A3 | 4 | |
| | B1 | 1 | |
| | B2 | 12 | |
| | B3 | 35 | |
| | L1 | 7 | |
| | L2 | 2 | |
| | L3 | 39 | |
| | p | 31 | |
| Indication for surgery | Strictures | 12 | |
| | Strictures & obstruction | 4 | |
| | Strictures & fistulae | 15 | |
| | Fistulae | 6 | |
| | Strictures &obstruction & fistulae | 5 | |
| | Fistulae & obstruction | 3 | |
| | Others | 3 | |
| Steroid | Yes | 17 | |
| | No | 31 | |
| Immunotherapy | Yes | 32 | |
| | No | 16 | |

[1]Limberg score:

Grade I: wall thickening (hypoechoic wall thickening and partially obscured mural stratification) and absent mural flow

Grade II : wall thickening with intermittent (or "spot") increases in vascularity

Grade III : wall thickening with protracted stretches of increased vascularity

Grade IV: color flow Doppler signals in both the bowel wall and surrounding mesenteric fat

[2] Montreal classification:

A1: below 16 y

A2: between 17 and 40 y

A3: above 40 y

B1: non-stricturing, non-penetrating

B2: stricturing

B3: penetrating

L1: ileal

L2: colonic

L3: ileocolonic

p: perianal disease modifier

Table S2

**Table S2. Primer sequences used in this study.**

| Origin | Primers | Forward | Reverse |
|---|---|---|---|
| **Mouse** | **IL-1β** | GCTGAAAGCTCTCCACCTCA | GCTTGGGATCCACACTCTCC |
| | **IL-6** | CTCTGCAAGAGACTTCCATCCA | GACAGGTCTGTTGGGAGTGG |
| | **TNF-α** | GCCTCTTCTCATTCCTGCTTG | CTGATGAGGGAGGCCATT |
| | **ZO-1** | TCATCCCAAATAAGAACAGAGC | GAAGAACAACCCTTTCATAAGC |
| | **Claudin1** | TGCCCCAGTGGAAGATTTACT | CTTTGCGAAACGCAGGACAT |
| | **Occludin** | TGAAAGTCCACCTCCTTACAGA | CCGGATAAAAAGAGTACGCTGG |
| | **Synpo** | ATGGAGGGGTACTCAGAGGAG | CTCTCGGTTTTGGGACAGGTG |
| | **Gapdh** | TGAAGCAGGCATCTGAGGG | CGAAGGTGGAAGAGTGGGAG |
| **Rat** | **IL-1β** | CACCTCTCAAGCAGAGCACAG | GGGTTCCATGGTGAAGTCAAC |
| | **IL-6** | AAAGAGTTGTGCAATGGCAATTCT | CAGTGCATCATCGCTGTTCATACA |
| | **TNF-α** | ACTGAACTTCGGGGTGATTG | GCTTGGTGGTTTGCTACGAC |
| | **Gapdh** | GGCATTGCTCTCAATGACAA | AGGGCCTCTCTCTTGCTCTC |
| ***K. variicola*** | | TACTTGTTCGACACGCGGAA, | CAATGGGCAACGAAAACGGT |
| ***ClpV*** | | ATGGGATATGGATGCGCTGT | AGGTGAACACTGCGGATCTG |
| **16S rRNA** | | GTG STG CAY GGY TGT CGT CA | ACG TCR TCC MCA CCT TCC TC |
| **16S rRNA (V1-V9)27F/1492R** | | AGAGTTTGATCCTGGCTCAG | TACGACTTAACCCCAATCGC |
| **N20-ClpV** | | CTGACTAGTCTCCATTGCGCGGGCACAGCGTTTTAGAGCTAGAAATAG | |

Table S3

**Table S3 The strains isolated from MATs of patients with CD and non-CD controls**

| **Strains isolated from ≥2 specimens of patients with CD** | **Family** | **CD8** | **CD10** | **CD12** | **CD13** | **CD16** | **CD14** | **CD17** | **CD7** | **CD5** | **CD6** | **CD9** | **CD3** | **CD4** | **CD1** |
|---|---|---|---|---|---|---|---|---|---|---|---|---|---|---|---|
| *Achromobacter deleyi* | *Alcaligenaceae* | 0 | 1 | 0 | 1 | 1 | 1 | 1 | 0 | 0 | 0 | 0 | 0 | 0 | 0 |
| *Achromobacter pulmonis* | *Alcaligenaceae* | 1 | 1 | 1 | 1 | 1 | 1 | 0 | 0 | 0 | 0 | 0 | 0 | 0 | 0 |
| *Devosia riboflavina* | *Devosiaceae* | 1 | 0 | 0 | 1 | 1 | 1 | 1 | 0 | 0 | 0 | 0 | 0 | 0 | 0 |
| *Enterococcus durans* | *Enterococcaceae* | 1 | 0 | 0 | 0 | 0 | 0 | 0 | 1 | 0 | 0 | 0 | 0 | 0 | 0 |
| *Enterococcus faecalis* | *Enterococcaceae* | 0 | 0 | 0 | 0 | 0 | 0 | 0 | 0 | 0 | 0 | 0 | 1 | 1 | 1 |
| *Escherichia/Shigella* | *Enterobacteriaceae* | 1 | 0 | 1 | 0 | 0 | 0 | 0 | 0 | 1 | 1 | 1 | 0 | 0 | 0 |
| *Klebsiella sp.* | *Enterobacteriaceae* | 1 | 0 | 0 | 0 | 0 | 0 | 0 | 0 | 0 | 0 | 0 | 1 | 1 | 1 |
| *Ochrobactrum anthropi* | *Brucellaceae* | 1 | 1 | 1 | 1 | 0 | 1 | 1 | 0 | 0 | 0 | 0 | 0 | 0 | 0 |
| *Pseudacidovorax intermedius* | *Comamonadaceae* | 0 | 0 | 0 | 1 | 1 | 0 | 1 | 0 | 0 | 0 | 0 | 0 | 0 | 0 |
| *Pseudomonas alcaliphila* | *Pseudomonadaceae* | 1 | 1 | 1 | 1 | 1 | 1 | 0 | 0 | 0 | 0 | 0 | 0 | 0 | 0 |
| *Staphylococcus capitis* | *Staphylococcaceae* | 0 | 0 | 0 | 0 | 0 | 0 | 0 | 0 | 0 | 0 | 0 | 0 | 0 | 0 |
| *Staphylococcus epidermidis* | *Staphylococcaceae* | 0 | 0 | 0 | 0 | 0 | 0 | 0 | 0 | 0 | 0 | 0 | 0 | 0 | 0 |
| *Staphylococcus haemolyticus* | *Staphylococcaceae* | 0 | 0 | 0 | 0 | 0 | 0 | 0 | 0 | 0 | 0 | 0 | 0 | 0 | 0 |
| *Staphylococcus hominis* | *Staphylococcaceae* | 0 | 1 | 1 | 0 | 0 | 0 | 0 | 0 | 0 | 0 | 0 | 0 | 0 | 0 |
| *Staphylococcus warneri* | *Staphylococcaceae* | 0 | 0 | 0 | 0 | 0 | 0 | 0 | 0 | 0 | 0 | 0 | 0 | 0 | 0 |
| *Stenotrophomonas maltophilia* | *Xanthomonadaceae* | 1 | 0 | 0 | 0 | 1 | 0 | 1 | 0 | 0 | 0 | 0 | 0 | 0 | 0 |
| **Strains isolated from ≥2 specimens of non-CD controls** | **Family** | **non-CD-1** | **non-CD-2** | **non-CD-3** | **non-CD-4** | **non-CD-5** | **non-CD-6** | **non-CD-7** | **non-CD-8** | **non-CD-9** | | | | | |
| *Staphylococcus capitis*strain JCM 2420 | *Staphylococcaceae* | 1 | 0 | 0 | 1 | 1 | 0 | 0 | 0 | 0 | | | | | |
| *Escherichia fergusonii*ATCC 35469 | *Enterobacteriaceae* | 0 | 0 | 1 | 0 | 0 | 0 | 0 | 0 | 1 | | | | | |

| | | | | | | | | | | |
|---|---|---|---|---|---|---|---|---|---|---|
| *Staphylococcus warneri* strain AW 25 | *Staphylococcaceae* | 0 | 1 | 0 | 0 | 1 | 0 | 0 | 1 | 0 |
| *Staphylococcus epidermidis* strain NBRC 100911 | *Staphylococcaceae* | 1 | 1 | 0 | 0 | 0 | 0 | 0 | 0 | 0 |
| *Staphylococcus hominis*strain DM 122 | *Staphylococcaceae* | 0 | 1 | 0 | 0 | 0 | 1 | 1 | 0 | 0 |
| *Staphylococcus haemolyticus* strain JCM 2416 | *Staphylococcaceae* | 0 | 0 | 0 | 0 | 0 | 0 | 1 | 1 | 0 |

3

**Table S4 The distribution of *Klebsiella variicola* in MAT from CD and non-CD**

| CD/non-CD patients | | CT | CFU/mg | Positive rate |
|---|---|---|---|---|
| **CD patients** | A10 | 23.55 | 66537 | 34.04% |
| | A12 | Undetermined | 0 | |
| | A14 | Undetermined | 0 | |
| | A15 | Undetermined | 0 | |
| | A16 | 26.87 | 7146 | |
| | A17 | 34.25 | 50 | |
| | A18 | 33.97 | 61 | |
| | A20 | Undetermined | 0 | |
| | A21 | Undetermined | 0 | |
| | A23 | 33.83 | 67 | |
| | A24 | Undetermined | 0 | |
| | A25 | 34.47 | 43 | |
| | A27 | Undetermined | 0 | |
| | A29 | Undetermined | 0 | |
| | A3 | Undetermined | 0 | |
| | A30 | Undetermined | 0 | |
| | A31 | Undetermined | 0 | |
| | A32 | Undetermined | 0 | |
| | A33 | 32.14 | 207 | |
| | A34 | Undetermined | 0 | |
| | A45 | 34.47 | 43 | |
| | A47 | Undetermined | 0 | |
| | A48 | Undetermined | 0 | |
| | A5 | Undetermined | 0 | |
| | A51 | Undetermined | 0 | |
| | A59 | 35.41 | 23 | |
| | A6 | Undetermined | 0 | |
| | A60 | Undetermined | 0 | |
| | A61 | Undetermined | 0 | |
| | A62 | 36.13 | 14 | |
| | A63 | 31.96 | 234 | |
| | A64 | Undetermined | 0 | |
| | A65 | 33.89 | 64 | |
| | A66 | Undetermined | 0 | |
| | A67 | Undetermined | 0 | |
| | A68 | 33.37 | 91 | |
| | A69 | Undetermined | 0 | |
| | A7 | Undetermined | 0 | |

| | | | | |
|---|---|---|---|---|
| | A70 | Undetermined | 0 | |
| | A71 | Undetermined | 0 | |
| | A72 | 32.00 | 228 | |
| | A73 | 29.98 | 883 | |
| | A74 | Undetermined | 0 | |
| | A75 | Undetermined | 0 | |
| | A76 | 33.97 | 61 | |
| | A8 | Undetermined | 0 | |
| | A9 | Undetermined | 0 | |
| | | | | |
| **non-CD patients** | C11 | Undetermined | 0 | 22.22% |
| | C12 | 29.433 | 7185 | |
| | C14 | 32.44 | 1243 | |
| | C15 | Undetermined | 0 | |
| | C16 | Undetermined | 0 | |
| | C17 | Undetermined | 0 | |
| | C18 | 31.452 | 2212 | |
| | C19 | Undetermined | 0 | |
| | C20 | 33.614 | 627 | |
| | C21 | Undetermined | 0 | |
| | C22 | Undetermined | 0 | |
| | C23 | Undetermined | 0 | |
| | C24 | Undetermined | 0 | |
| | C25 | Undetermined | 0 | |
| | C26 | Undetermined | 0 | |
| | C27 | Undetermined | 0 | |
| | C28 | Undetermined | 0 | |
| | C29 | Undetermined | 0 | |

**Table S5 The distribution of *Klebsiella variicol*a in the mucosal biopsies from CD and non-CD**

| **CD/non-CD patients** | | **CT** | **CFU/mg** | **Positive rate** |
|---|---|---|---|---|
| CD patients | Ac1 | 34.29 | 122 | 43.75% |
| | Ac2 | 34.42 | 112 | |
| | Ac3 | Undetermined | 0 | |
| | Ac4 | 33.45 | 215 | |
| | Ac5 | 38.15 | 9 | |
| | Ac6 | Undetermined | 0 | |
| | Ac7 | Undetermined | 0 | |
| | Ac8 | Undetermined | 0 | |

| | | | | |
|---|---|---|---|---|
| | Ac9 | Undetermined | 0 | |
| | Ac10 | Undetermined | 0 | |
| | Ac11 | Undetermined | 0 | |
| | Ac12 | Undetermined | 0 | |
| | Ac13 | Undetermined | 0 | |
| | Ac14 | 27.27 | 13718 | |
| | Ac15 | 35.13 | 69 | |
| | Ac16 | 37.53 | 14 | |
| | | | | |
| **non-CD patients** | Cc1 | 36.37 | 30 | 12.50% |
| | Cc2 | Undetermined | 0 | |
| | Cc3 | Undetermined | 0 | |
| | Cc4 | Undetermined | 0 | |
| | Cc5 | Undetermined | 0 | |
| | Cc6 | Undetermined | 0 | |
| | Cc7 | Undetermined | 0 | |
| | Cc8 | Undetermined | 0 | |

## Table S6 Genes assigned to type VI secretion system

| #GeneID | start | end | Gene length | | KEGG_annotation |
|---|---|---|---|---|---|
| **GE00840** | 892204 | 892695 | 491 | + | K11901\|8.41309e-106\|kpt:VK055_1132\|hypothetical protein; K11901 type VI secretion system protein ImpB (A) |
| **GE00860** | 909497 | 909928 | 431 | + | K11901\|1.95084e-85\|kvd:KR75_19315\|type VI secretion protein; K11901 type VI secretion system protein ImpB (A) |
| **GE00861** | 910030 | 911574 | 1544 | + | K11900\|0\|kvd:KR75_19320\|EvpB family type VI secretion protein; K11900 type VI secretion system protein ImpC (A) |
| **GE00862** | 911584 | 912927 | 1343 | + | K11893\|0\|kpk:A593_21455\|hypothetical protein; K11893 type VI secretion system protein ImpJ (A) |
| **GE00863** | 912924 | 913613 | 689 | + | K11892\|1.44184e-165\|kva:Kvar_2972\|type IV / VI secretion system protein, DotU family; K11892 type VI secretion system protein ImpK (A) |
| **GE00865** | 915318 | 915809 | 491 | + | K11903\|7.81129e-118\|kps:KPNJ2_03095\|hypothetica l protein; K11903 type VI secretion system secreted protein Hcp (A) |
| **GE00866** | 916074 | 918728 | 2654 | + | K11907\|0\|kpe:KPK_3063\|clpV; type VI secretion ATPase, ClpV1 family; K11907 type VI secretion system protein VasG (A) |

| | | | | | |
|---|---|---|---|---|---|
| **GE00867** | 918721 | 921099 | 2378 | + | K11904\|0\|kva:Kvar_2968\|Rhs element Vgr protein; K11904 type VI secretion system secreted protein VgrG (A) |
| **GE00879** | 931757 | 935185 | 3428 | + | K11891\|0\|kva:Kvar_2958\|ImcF domain-containing protein; K11891 type VI secretion system protein ImpL (A) |
| **GE00880** | 935182 | 936774 | 1592 | + | K11910\|0\|kpt:VK055_1092\|impA-related N-terminal family protein; K11910 type VI secretion system protein VasJ (A) |
| **GE00881** | 936854 | 938608 | 1754 | + | K11896\|0\|kvd:KR75_19380\|type VI secretion protein ImpG; K11896 type VI secretion system protein ImpG (A) |
| **GE00882** | 938572 | 939657 | 1085 | + | K11895\|0\|kpk:A593_21565\|hypothetical protein; K11895 type VI secretion system protein ImpH (A) |
| **GE00883** | 939635 | 940177 | 542 | + | K11906\|1.5951e-126\|kva:Kvar_2954\|type VI secretion lipoprotein; K11906 type VI secretion system protein VasD (A) |
| **GE01790** | 1875806 | 1877191 | 1385 | - | K11911\|0\|kpe:KPK_2057\|ImpA domain-containing protein; K11911 type VI secretion system protein VasL (A) |
| **GE01791** | 1877211 | 1877654 | 443 | - | K11905\|1.25542e-101\|kvd:KR75_24595\|type VI secretion system lysozyme-like protein; K11905 type VI secretion system protein (A) |
| **GE01792** | 1877657 | 1878193 | 536 | - | K11906\|3.97974e-126\|kpu:KP1_3367\|hypothetical protein; K11906 type VI secretion system protein VasD (A) |
| **GE01793** | 1878174 | 1879220 | 1046 | - | K11895\|0\|kpk:A593_00820\|type VI secretion protein; K11895 type VI secretion system protein ImpH (A) |
| **GE01794** | 1879220 | 1880983 | 1763 | - | K11896\|0\|kpn:KPN_02250\|hypothetical protein; K11896 type VI secretion system protein ImpG (A) |
| **GE01795** | 1881118 | 1884513 | 3395 | - | K11891\|0\|kpk:A593_00830\|type VI secretion protein VasK; K11891 type VI secretion system protein ImpL (A) |
| **GE01804** | 1894785 | 1897484 | 2699 | - | K11904\|0\|kpt:VK055_0186\|vgrG2; valine-glycine repeat protein G; K11904 type VI secretion system secreted protein VgrG (A) |
| **GE01806** | 1899647 | 1900300 | 653 | - | K11892\|9.64329e-157\|kpe:KPK_2042\|type IV/VI secretion system protein, DotU family; K11892 type VI secretion system protein ImpK (A) |
| **GE01807** | 1900297 | 1901637 | 1340 | - | K11893\|0\|kpk:A593_00885\|hypothetical protein; K11893 type VI secretion system protein ImpJ (A) |
| **GE02708** | 2876318 | 2876824 | 506 | + | K11903\|2.23457e-123\|kpx:PMK1_00572\|hcpA_1; Secreted protein hcp; K11903 type VI secretion |

| | | | | | |
|---|---|---|---|---|---|
| | | | | | system secreted protein Hcp (A) |
| **GE04306** | 4604093 | 4604578 | 485 | + | K11903\|1.5935e-109\|ror:RORB6_16040\|type VI secretion system effector; K11903 type VI secretion system secreted protein Hcp (A) |

**Table S7. Six representative genes of T6SS in *K. variicola* ZSLY01**

| **#GeneID** | **Gene length** | **PCR product length** | **KEGG_annotation** |
|---|---|---|---|
| **GE00865** | 491 bp | 170 bp | K11903\|7.81129e-118\|kps:KPNJ2_03095\|hypothetical protein; K11903 type VI secretion system secreted protein Hcp |
| **GE00866** | 2654 bp | 910 bp | K11907\|0\|kpe:KPK_3063\|clpV; type VI secretion ATPase, ClpV1 family; K11907 type VI secretion system protein VasG |
| **GE00867** | 2378 bp | 362 bp | K11904\|0\|kva:Kvar_2968\|Rhs element Vgr protein; K11904 type VI secretion system secreted protein VgrG |
| **GE01804** | 2699 bp | 212 bp | K11904\|0\|kpt:VK055_0186\|vgrG2; valine-glycine repeat protein G; K11904 type VI secretion system secreted protein VgrG |
| **GE02708** | 506 bp | 298 bp | K11903\|2.23457e-123\|kpx:PMK1_00572\|hcpA_1; Secreted protein hcp; K11903 type VI secretion system secreted protein Hcp |
| **GE04306** | 485 bp | 295 bp | K11903\|1.5935e-109\|ror:RORB6_16040\|type VI secretion system effector; K11903 type VI secretion system secreted protein Hcp |
